# Precision Perioperative Treatment for Resectable Non-Small Cell Lung Cancer: Current Status and Perspectives

**DOI:** 10.3390/cancers18213456

**Published:** 2026-10-26

**Authors:** Dayna J. Isaacs, Cathleen J. Park, Linjun Zha, Ruqiang Liang, Luis A. Godoy, Nicholas S. Stollenwerk, Dongguang Wei, Kao Y. Chang, Prabhu Rajappa, Tianhong Li

**Affiliations:** 1 Division of Hematology/Oncology, Department of Internal Medicine, University of California Comprehensive Cancer Center, Sacramento, CA 95817, USAlzha@health.ucdavis.edu (L.Z.);; 2 Medical Service, Veterans Affairs Northern California Health Care System, Mather, CA 95655, USAprabhu.rajappa@va.gov (P.R.); 3 Division of Thoracic Surgery, Department of Surgery, University of California Davis School of Medicine, Sacramento, CA 95817, USA; lagodoy@health.ucdavis.edu; 4 Surgery Service, Veterans Affairs Northern California Health Care System, Mather, CA 95655, USA; 5 Division of Pulmonary, Critical Care, and Sleep Medicine, Department of Internal Medicine, University of California Davis School of Medicine, Sacramento, CA 95817, USA; 6 Department of Pathology & Laboratory Medicine, University of California Davis School of Medicine, Sacramento, CA 95817, USA; donwei@health.ucdavis.edu; 7 Investigational Drugs Pharmacy Program, Veterans Affairs Northern California Health Care System, Mather, CA 95655, USA; kao.chang@va.gov

**Keywords:** non-small cell lung cancer, precision oncology, neoadjuvant therapy, perioperative therapy, chemoimmunotherapy, targeted therapy, oncogenic drivers, actionable genomic alteration, biomarkers

## Abstract

The treatment paradigm for resectable non-small cell lung cancer (NSCLC) continues to evolve toward precision perioperative strategies. Despite curative-intent surgery, disease recurrence remains common. Adjuvant tyrosine kinase inhibitors have substantially improved disease-free survival and, in selected populations, overall survival, particularly for patients with sensitizing *EGFR* mutations or *ALK* rearrangements. Targeted therapies for other actionable genomic alterations (AGAs), including *RET* fusions, remain investigational. Adequate tissue acquisition and comprehensive molecular profiling before treatment initiation are therefore essential for optimal treatment selection. For patients without sensitizing *EGFR* mutations or *ALK* rearrangements, neoadjuvant or perioperative PD-(L)1 blockade combined with platinum-based chemotherapy has become a standard of care for eligible patients with resectable stage II–III NSCLC, improving pathological response, event-free survival, and, in select trials, overall survival. Multidisciplinary reassessment of resectability following neoadjuvant treatment is particularly important for borderline-resectable disease. Patients subsequently deemed unresectable require individualized management, potentially including definitive chemoradiotherapy followed by consolidation immunotherapy. Although cross-trial comparisons suggest advantages of neoadjuvant chemoimmunotherapy over adjuvant-only immunotherapy, direct randomized comparisons are lacking. The incremental benefit of postoperative immunotherapy after neoadjuvant chemoimmunotherapy remains uncertain for selected patients. Future priorities include optimizing treatment sequencing, improving pathological complete response rates, refining radiographic and molecular response assessment, investigating resistance mechanisms, and integrating circulating tumor DNA-based minimal residual disease detection into individualized postoperative treatment. Whether surgery can be safely omitted in exceptional responders also warrants prospective investigation. Together, these advances support a biomarker-driven, response-adapted approach to precision perioperative NSCLC management.

## Simple Summary

The management of resectable non-small cell lung cancer (NSCLC) has shifted toward personalized perioperative treatment guided by tumor biology and treatment response. Adjuvant targeted therapies have improved outcomes for patients with classic sensitizing *EGFR* mutations and *ALK* rearrangements, and likely *RET* fusions, while neoadjuvant or perioperative PD-(L)1 blockade combined with platinum-based chemotherapy has become a standard of care for patients without actionable *EGFR* or *ALK* alterations. Adequate tissue acquisition and comprehensive tumor molecular profiling are therefore essential for treatment planning. Ongoing studies are investigating optimal treatment selection and sequencing, radiographic and molecular biomarkers, mechanisms of treatment resistance, blood circulating tumor DNA (ctDNA)-based detection of minimal residual disease, and individualized postoperative strategies. This review integrates current evidence into a practical treatment algorithm incorporating oncogenic driver status, resectability, treatment eligibility, and postoperative findings. Future biomarker-guided approaches may enable treatment de-escalation in selected patients and help define whether surgery can be safely omitted in exceptional responders.

## 1. Introduction

Lung cancer remains the leading cause of cancer-related mortality in the United States and worldwide, accounting for approximately 11% of new cancer diagnoses and 20% of cancer deaths in 2025 [1]. Non-small cell lung cancer (NSCLC) represents approximately 85% of cases, yet only 20–25% of patients are diagnosed with resectable disease [1]. Even after curative-intent surgery, recurrence remains common, with 5-year recurrence-free survival ranging from approximately 33% to 87%, depending largely on disease stage [1]. The widespread adoption of smoking cessation, lung cancer screening, and improved lung nodule surveillance has shifted diagnosis toward earlier-stage disease. Between 2010 and 2017, the incidence of stage I NSCLC increased while stage IV disease decreased [2]. Similarly, U.S. Cancer Statistics data showed that the proportion of lung cancers diagnosed at a localized stage increased from 17.6% in 2003 to 30.1% in 2022, with marked acceleration after 2014 [3]. This stage migration is clinically important because outcomes remain strongly stage-dependent, with 5-year overall survival (OS) rates of approximately 82% for stage IA, 66% for stage IB, 47–52% for stage II, and 19–36% for stage III disease [4].

Perioperative systemic therapy has long been investigated to reduce recurrence and improve survival in resectable NSCLC. Early randomized trials demonstrated a modest OS benefit with neoadjuvant chemotherapy, increasing 5-year survival from approximately 40% to 45% compared with surgery alone [5]. Subsequent trials, including NATCH and the Scagliotti et al. study, further established the feasibility and potential efficacy of preoperative systemic therapy [6,7]. With increasingly effective neoadjuvant treatments, pathologic response has emerged as an important intermediate endpoint. Pathologic complete response (pCR), defined as no residual viable tumor in the primary tumor and sampled lymph nodes, and major pathologic response (MPR), defined as ≤10% residual viable tumor in the primary tumor, are now routinely incorporated into neoadjuvant trials following the 2020 International Association for the Study of Lung Cancer recommendations [8].

Despite these advances, recurrence remains a major challenge, particularly in patients with stage II–III disease [9,10,11]. Since 2022, the perioperative treatment paradigm has expanded beyond cytotoxic chemotherapy to incorporate PD-(L)1 immune checkpoint inhibitors (ICIs) and genotype-directed targeted therapies [12,13,14,15,16]. For patients without actionable genomic alterations (AGAs), neoadjuvant and perioperative chemoimmunotherapy have become standards of care, while adjuvant targeted therapies have improved outcomes in selected oncogene-driven NSCLC, including classic sensitizing *EGFR* mutations and *ALK* rearrangements, and likely *RET* fusions. These advances have transformed resectable NSCLC into an increasingly biomarker-defined disease and underscore the importance of adequate tissue acquisition and comprehensive molecular profiling before treatment initiation. This review summarizes contemporary precision perioperative strategies for resectable NSCLC, including chemoimmunotherapy, genotype-directed targeted therapy, biomarkers of treatment response and recurrence, and evolving approaches to individualize postoperative management.

## 2. Perioperative PD-(L)1 Inhibitors for NSCLC Without AGAs

### 2.1. Nivolumab 

#### 2.1.1. CheckMate-816

Nivolumab plus platinum-based chemotherapy was the first ICI regimen to receive U.S. Food and Drug Administration (FDA) approval, in March 2022, for neoadjuvant treatment of resectable NSCLC. The phase III CheckMate-816 trial randomized 358 patients with untreated, resectable stage IB–IIIA NSCLC (AJCC 7th edition) to three cycles of neoadjuvant nivolumab plus platinum-doublet chemotherapy or chemotherapy alone, followed by curative-intent surgery [17]. Surgery was performed in 83.2% and 75.4% of patients, respectively.

CheckMate-816 met both primary endpoints, with nivolumab plus chemotherapy significantly increasing pCR compared with chemotherapy alone (24.0% vs. 2.2%; *p* < 0.001) [17]. At the 5-year follow-up, neoadjuvant nivolumab plus chemotherapy continues to demonstrate a significant event-free survival (EFS) benefit with a median EFS of 59.6 versus 21.1 months with neoadjuvant chemotherapy (HR, 0.68; 95% CI, 0.51–0.91) and 5-year EFS rates of 49% versus 34%, respectively [18]. The final analysis also demonstrated a significant OS benefit with 5-year OS rates of 65.4% versus 55.0% (HR 0.72; 95% CI, 0.523–0.998; *p* = 0.048) [18]. Exploratory analyses further identified potential biomarkers of response. ctDNA clearance was more frequent with nivolumab plus chemotherapy than with chemotherapy alone (56% vs. 35%) and was associated with higher pCR rates and improved EFS and OS [17,18]. An exploratory nivolumab-plus-ipilimumab cohort also had higher pCR and MPR rates than chemotherapy alone, with a four-gene inflammatory signature (CD8A, STAT1, LAG3, and CD274) associated with improved outcomes [19]. 

Collectively, CheckMate-816 established neoadjuvant chemoimmunotherapy as a standard-of-care treatment option for eligible patients with resectable NSCLC. 

#### 2.1.2. NADIM II

The phase II NADIM II trial randomized 86 patients with resectable stage III NSCLC (AJCC 7th edition) to three cycles of neoadjuvant nivolumab plus carboplatin and paclitaxel or chemotherapy alone [20]. Nivolumab plus chemotherapy significantly increased the primary endpoint of pCR (37% vs. 7%) and improved 2-year PFS (67.2% vs. 40.9%; HR, 0.47) and OS (85.0% vs. 63.6%; HR, 0.43). Higher pCR rates were observed in patients with PD-L1 expression ≥1%. Exploratory analyses demonstrated greater ctDNA clearance with chemoimmunotherapy (67% vs. 44%), consistent with findings from CheckMate-816 [21].

Unlike CheckMate-816, which was an international trial, NADIM II was conducted exclusively in Spain and incorporated six months of adjuvant nivolumab following R0 resection. Among eligible patients, 84% received at least one cycle and 66% completed adjuvant treatment. Post hoc analyses suggested favorable PFS and OS; however, because receipt and completion of postoperative therapy were not independently randomized, the specific contribution of adjuvant nivolumab cannot be determined from NADIM II.

#### 2.1.3. CheckMate-77T

The phase III CheckMate-77T trial randomized 461 patients with resectable stage IIA–IIIB (T3N2, AJCC 8th edition) NSCLC to perioperative nivolumab—neoadjuvant nivolumab plus chemotherapy followed by surgery and adjuvant nivolumab—or neoadjuvant chemotherapy followed by surgery and adjuvant placebo [22]. Definitive surgery was performed in 78% and 77% of patients, respectively. With a median follow-up of 41.0 months, perioperative nivolumab improved EFS, with a median EFS of 46.6 months versus 16.9 months with chemotherapy/placebo (HR, 0.61; 95% CI, 0.46–0.80) [23]. At the first prespecified interim OS analysis, the 30-month OS rate was 78% versus 72% (HR, 0.85; 95% CI, 0.61–1.18). Although the difference did not reach statistical significance [23], benefits were observed across disease stage, histology, and PD-L1 subgroups [22,23].

Exploratory analyses demonstrated substantial benefit among patients with stage III and N2 disease [24]. Among patients with N2 involvement, perioperative nivolumab improved 1-year EFS (70% vs. 45%; HR, 0.46) and pCR (22.0% vs. 5.6%), with benefit also observed in multi-station N2 disease [24]. These findings support consideration of multimodality treatment, including surgery, for appropriately selected patients with resectable N2 disease. A key distinction from CheckMate-816 is the incorporation of adjuvant nivolumab, although the magnitude of benefit attributable specifically to the postoperative component remains uncertain.

#### 2.1.4. Nivolumab Summary

**Figure 1:**
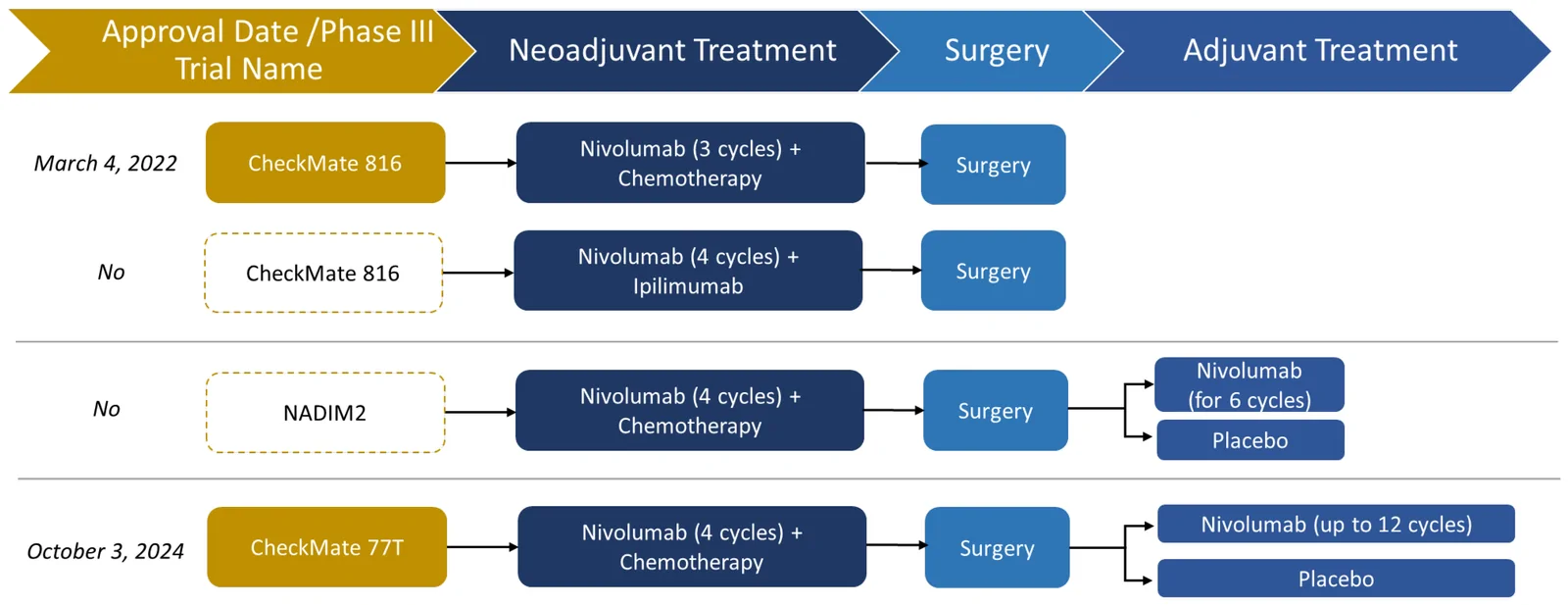
Duration and timing of perioperative nivolumab therapy in resectable early-stage NSCLC. Schematic comparison of clinical trial strategies evaluating nivolumab-based therapy for resectable NSCLC, illustrating differences in the timing and duration of immune checkpoint inhibition. Neoadjuvant-only strategies administer nivolumab with platinum-based chemotherapy before surgery without routine postoperative nivolumab, whereas perioperative strategies extend nivolumab treatment after surgery for a protocol-defined duration. Abbreviations: ICI, immune checkpoint inhibitor; NSCLC, non-small cell lung cancer.

Collectively, CheckMate-816, NADIM II, and CheckMate-77T established nivolumab-based chemoimmunotherapy as an effective strategy for resectable NSCLC (Figure 1), consistently improving pCR and EFS compared with chemotherapy alone, with a confirmed OS benefit demonstrated in CheckMate-816. CheckMate-77T has demonstrated a statistically significant EFS benefit, but not yet a statistically significant OS benefit, unlike the more mature CheckMate-816 OS results. This figure highlights the substantial variation in total nivolumab exposure across clinical trials and the unresolved question of the incremental benefit and optimal duration of postoperative immune checkpoint inhibition following effective neoadjuvant chemoimmunotherapy and complete surgical resection. Although greater benefit has generally been observed in tumors with PD-L1 expression ≥1% and more advanced resectable disease, clinically meaningful responses also occur in patients with PD-L1-negative tumors.

Several questions remain regarding optimal patient selection and treatment duration. In particular, the incremental benefit of adjuvant immunotherapy following effective neoadjuvant chemoimmunotherapy remains incompletely defined because CheckMate-77T compared a perioperative strategy with neoadjuvant chemotherapy rather than directly randomizing patients after surgery to adjuvant nivolumab or observation. Whether pCR can identify patients who may safely omit additional postoperative therapy, and whether patients without pCR require treatment escalation, remain important areas of investigation. Similarly, perioperative ctDNA clearance and postoperative molecular residual disease (MRD) are emerging as promising markers of treatment response and recurrence risk and may ultimately help guide individualized adjuvant treatment.

### 2.2. Pembrolizumab

#### 2.2.1. KEYNOTE-091

The benefit of pembrolizumab in resectable NSCLC was demonstrated in the adjuvant setting in the phase III PEARLS/KEYNOTE-091 trial [25]. A total of 1177 patients with completely resected stage IB (tumor ≥ 4 cm)–IIIA NSCLC (AJCC TNM 7th edition), regardless of PD-L1 expression, were randomized to receive pembrolizumab or placebo for up to 1 year [25]. Adjuvant chemotherapy was strongly recommended for patients with stage II–IIIA disease and considered for those with stage IB disease but was not required; overall, 86% of patients received adjuvant platinum-based chemotherapy [25].

At the final DFS analysis, after a median follow-up of 51.7 months, pembrolizumab improved DFS in the overall population, with a median DFS of 53.8 versus 43.0 months (HR, 0.81; 95% CI, 0.68–0.96) [26]. In the prespecified PD-L1 TPS ≥ 50% population, median DFS was 67.0 versus 47.6 months (HR, 0.83; 95% CI, 0.59–1.16; *p* = 0.13), and the co-primary DFS endpoint in this subgroup was not met [27]. Among patients who received adjuvant chemotherapy, median DFS was 53.8 versus 40.5 months (HR, 0.80; 95% CI, 0.67–0.96) [27]. OS data remain immature, and a statistically significant OS benefit with adjuvant pembrolizumab has not yet been established.

#### 2.2.2. KEYNOTE-671

The phase III KEYNOTE-671 trial subsequently evaluated perioperative pembrolizumab in 797 patients with resectable stage II–IIIB NSCLC (AJCC 8th edition) [28]. Patients were randomized to four cycles of neoadjuvant pembrolizumab plus platinum-based chemotherapy followed by surgery and up to 13 cycles of adjuvant pembrolizumab, or neoadjuvant chemotherapy followed by surgery and adjuvant placebo. Unlike several neoadjuvant immunotherapy trials, KEYNOTE-671 did not exclude patients with known *EGFR* mutations or *ALK* rearrangements; however, these subgroups were small (33 and 21 patients, respectively), limiting conclusions regarding efficacy in oncogene-driven NSCLC.

Perioperative pembrolizumab significantly increased pCR (18.1% vs. 4.0%) and MPR (30.2% vs. 11.0%) [28]. With 5 years of follow-up, median EFS was 57.1 versus 18.4 months (HR, 0.58; 95% CI, 0.48–0.69), with 5-year EFS rates of 49.9% versus 26.5%. Five-year OS rates were 64.6% versus 53.6% (HR, 0.74; 95% CI, 0.59–0.92) [29]. Benefit was maintained across disease-stage and PD-L1 subgroups, although the magnitude of benefit was numerically smaller in patients with PD-L1 TPS < 1% [29]. The trial was not designed to distinguish the relative contributions of neoadjuvant versus adjuvant pembrolizumab. Based on KEYNOTE-671, the FDA approved perioperative pembrolizumab in October 2023 for resectable NSCLC with tumors ≥ 4 cm or node-positive disease.

### 2.3. Atezolizumab

#### 2.3.1. IMpower010

In October 2021, atezolizumab became the first ICI to receive FDA approval for adjuvant treatment of resected NSCLC based on the phase III IMpower010 trial [30]. Patients with completely resected stage IB (tumor ≥ 4 cm)–IIIA NSCLC (AJCC 7th edition) received platinum-based adjuvant chemotherapy and were subsequently randomized to up to 16 cycles of atezolizumab or best supportive care. Atezolizumab significantly improved disease-free survival (DFS) in patients with stage II–IIIA disease (HR, 0.79; 95% CI, 0.64–0.96; *p* = 0.020), with greater benefit among patients with PD-L1 expression ≥ 1% (HR, 0.66; 95% CI, 0.50–0.88; *p* = 0.0039). These findings led to FDA approval of adjuvant atezolizumab following resection and platinum-based chemotherapy for patients with stage II–IIIA NSCLC with PD-L1 expression on ≥1% of tumor cells [30]. At the final DFS analysis after ≥5 years of follow-up, the benefit of atezolizumab was sustained in the stage II–IIIA PD-L1 TC ≥ 1% population (median DFS, 68.5 vs. 37.3 months; HR, 0.70; 95% CI, 0.55–0.91), with a more pronounced benefit in patients with PD-L1 TC ≥ 50% (median DFS not reached vs. 41.1 months; HR, 0.48; 95% CI, 0.32–0.72) [31]. OS results showed a similar relationship with PD-L1 expression. No OS benefit was observed in the overall stage II–IIIA population (HR, 0.94; 95% CI, 0.75–1.19), whereas a favorable OS trend was observed among patients with PD-L1 TC ≥ 1% (HR, 0.77; 95% CI, 0.56–1.06; 5-year OS, 74.8% vs. 66.3%). The greatest OS benefit was observed in patients with PD-L1 TC ≥ 50% (HR, 0.47; 95% CI, 0.28–0.77; 5-year OS, 82.7% vs. 65.3%) [31]. Although these subgroup OS analyses were not formally tested within the hierarchical statistical testing strategy, the long-term results suggest that the magnitude of benefit from adjuvant atezolizumab is greatest in tumors with high PD-L1 expression.

#### 2.3.2. LCMC3 and Other Neoadjuvant Atezolizumab Studies

The phase II, single-arm LCMC3 trial evaluated two cycles of neoadjuvant atezolizumab monotherapy in 181 patients with resectable stage IB–IIIB NSCLC (AJCC 8th edition), followed by surgery and optional standard adjuvant therapy [32,33]. The trial achieved an MPR rate of 20% and a pCR rate of 6% [32]. A long-term follow-up analysis published in 2024 reported 4-year DFS and OS rates of 70% and 82%, respectively [34]. Although patients who received adjuvant atezolizumab appeared to have improved outcomes, interpretation is limited by the nonrandomized design and potential selection bias.

A separate phase II study by Shu et al. evaluated neoadjuvant atezolizumab combined with carboplatin and nab-paclitaxel in 30 patients with resectable stage IB–IIIA NSCLC [35]. R0 resection was achieved in 87% of patients and MPR in 57%, providing early evidence supporting the combination of atezolizumab with neoadjuvant chemotherapy.

#### 2.3.3. IMpower030

The phase III IMpower030 trial evaluated perioperative atezolizumab in patients with resectable stage II–IIIA and selected stage IIIB (T3N2) NSCLC (AJCC 8th edition). A total of 440 patients were randomized to four cycles of neoadjuvant atezolizumab or placebo combined with platinum-doublet chemotherapy, followed by surgery and up to 16 cycles of adjuvant atezolizumab in the experimental arm [36]. The primary endpoint was EFS, with pCR and MPR among the key secondary endpoints. Final results presented at the 2026 World Conference on Lung Cancer (WCLC) showed that IMpower030 did not meet its primary EFS endpoint [37]. In the primary stage IIB–IIIB *EGFR*/*ALK* wild-type population, median EFS was 62.8 months with atezolizumab versus 34.9 months with placebo (HR, 0.77; 95% CI, 0.58–1.02; *p* = 0.07) [37]. Despite the statistically negative primary endpoint, pathological responses favored atezolizumab; in the broader intention-to-treat *EGFR*/*ALK* wild-type population, pCR rates were 30.6% versus 8.6%, and MPR rates were 54.3% versus 24.9%, respectively [38]. OS also numerically favored atezolizumab but was not statistically significant. Thus, IMpower030 should be considered a statistically negative phase III trial despite numerical improvements across several efficacy endpoints. Importantly, these findings should not alter the established role of neoadjuvant or perioperative chemoimmunotherapy in appropriately selected patients with resectable NSCLC, given the positive results of other pivotal phase III trials. 

### 2.4. Durvalumab

Durvalumab was initially established as consolidation therapy for unresectable stage III NSCLC following chemoradiation in the PACIFIC trial [39]. Its role subsequently expanded into resectable disease through SAKK 16/14 and the phase III AEGEAN trial, ultimately leading to FDA approval of perioperative durvalumab in 2024 [40,41].

#### 2.4.1. PACIFIC

The phase III PACIFIC trial randomized 713 patients with unresectable stage III NSCLC without progression following concurrent chemoradiation to durvalumab or placebo for up to 12 months. Durvalumab significantly improved both co-primary endpoints of PFS and OS. At the 5-year update, median PFS was 16.9 versus 5.6 months (HR, 0.55; 95% CI, 0.45–0.68), and median OS was 47.5 versus 29.1 months (HR, 0.72; 95% CI, 0.59–0.89) [42]. Five-year PFS rates were 33.1% versus 19.0%, and 5-year OS rates were 42.9% versus 33.4%, respectively [42]. These durable survival benefits established consolidation durvalumab following concurrent chemoradiotherapy as a standard of care for unresectable stage III NSCLC and provided a foundation for its subsequent investigation in earlier-stage disease.

#### 2.4.2. SAKK 16/14

The phase II SAKK 16/14 trial evaluated perioperative durvalumab in 68 patients with resectable stage IIIA(N2) NSCLC (AJCC 7th edition) [40]. Patients received three cycles of neoadjuvant platinum-based chemotherapy followed by two cycles of durvalumab and surgery, with adjuvant durvalumab administered for up to one year. Among 55 patients who underwent surgery, 93% achieved R0 resection, and 62% achieved major pathologic response (MPR) [40]. The trial met its primary endpoint, with a 1-year EFS rate of 73%, providing early evidence supporting perioperative durvalumab in resectable stage III disease. With longer follow-up of 5.4 years, median EFS was 4.0 years and median OS was not reached; 5-year EFS and OS rates were 46% and 66%, respectively [43]. These findings demonstrated durable outcomes with sequential neoadjuvant chemotherapy and perioperative durvalumab in resectable stage IIIA(N2) disease.

#### 2.4.3. AEGEAN

The phase III AEGEAN trial randomized 802 patients with resectable stage II–IIIB (N2) NSCLC (AJCC 8th edition) to four cycles of neoadjuvant durvalumab plus platinum-based chemotherapy followed by surgery and up to 12 cycles of adjuvant durvalumab, or chemotherapy followed by surgery and placebo [44]. Perioperative durvalumab significantly increased pCR from 4.3% to 17.2% (*p* < 0.001) [44]. At the second planned interim analysis, the EFS benefit was maintained (HR, 0.69; 95% CI, 0.55–0.88), with median EFS not reached versus 30.0 months [45]. DFS among resected patients also favored durvalumab (HR, 0.66; 95% CI, 0.47–0.92), while interim OS showed a numerical but not definitive improvement (HR, 0.89; 95% CI, 0.70–1.14). Benefits were observed across stage, histology, and PD-L1 subgroups, with a numerically greater magnitude of benefit in tumors with PD-L1 expression ≥ 50%, although no significant treatment-by-subgroup interaction was identified. Surgical feasibility was maintained with perioperative durvalumab, with similar proportions of patients proceeding to surgery and a slightly higher R0 resection rate observed compared with the control arm (94.7% vs. 91.3%) [46]. Based on AEGEAN, the FDA approved neoadjuvant durvalumab plus platinum-containing chemotherapy followed by adjuvant durvalumab in August 2024 for adults with resectable NSCLC (tumors ≥4 cm and/or node-positive) without known *EGFR* exon 19 deletions or exon 21 L858R mutations or *ALK* rearrangements. OS data remain immature. 

Correlative analyses have further highlighted the potential role of ctDNA as a biomarker. Postoperative ctDNA-defined MRD positivity was strongly associated with inferior outcomes, supporting the prognostic value of perioperative ctDNA and its potential future role in risk-adapted postoperative treatment [47].

### 2.5. Toripalimab

#### NEOTORCH

The phase III NEOTORCH trial evaluated perioperative toripalimab, a PD-1 inhibitor, in combination with chemotherapy for resectable stage II–IIIB NSCLC (AJCC TNM 8th edition) [48]. A total of 501 patients were randomized to four cycles of neoadjuvant toripalimab plus platinum-doublet chemotherapy or chemotherapy plus placebo, followed by surgery and 13 cycles of adjuvant toripalimab or placebo. The study population was predominantly male (91.6%), with squamous histology (77.7%) and stage IIIA disease (67.3%), which should be considered when assessing generalizability.

At a median follow-up of 18.3 months, perioperative toripalimab significantly improved EFS (median not reached vs. 15.1 months; HR, 0.40; 95% CI, 0.28–0.57; *p* < 0.0001), MPR (48.5% vs. 8.4%), and pCR (24.8% vs. 1.0%) [48]. EFS benefit was observed across PD-L1 expression subgroups and was particularly pronounced in squamous NSCLC, whereas benefit in the smaller nonsquamous subgroup was less certain. OS also favored toripalimab but did not reach statistical significance at the interim analysis (HR, 0.62; 95% CI, 0.38–0.999; *p* = 0.0502) [48]. Longer follow-up is needed to define the mature survival benefit and efficacy across histologic subgroups.

### 2.6. Penpulimab

#### ALTER-L043

ALTER-L043 is a randomized phase II trial evaluating PD-1 blockade combined with the oral anti-angiogenic agent anlotinib in resectable stage IIB–IIIB NSCLC [49]. Ninety patients were randomized 1:1:1 to receive neoadjuvant penpulimab plus anlotinib and chemotherapy, penpulimab plus chemotherapy, or penpulimab plus anlotinib, followed by surgery and corresponding adjuvant therapy. The triplet regimen produced the highest MPR rate (76.0% vs. 57.7% and 52.4%, respectively), although the differences were not statistically significant. pCR rates were 52.0%, 50.0%, and 38.1%, respectively. Definitive surgery was performed in 92.6%, 89.7%, and 70.0% of patients. At a median follow-up of 14.0 months, median EFS and OS were not reached in any group; 12-month EFS rates were 94.1%, 89.3%, and 73.6%, respectively. These findings provide preliminary evidence supporting the feasibility of incorporating anti-angiogenic therapy into perioperative chemoimmunotherapy, but larger randomized studies with longer follow-up are needed to establish its clinical benefit.

**Figure 2:**
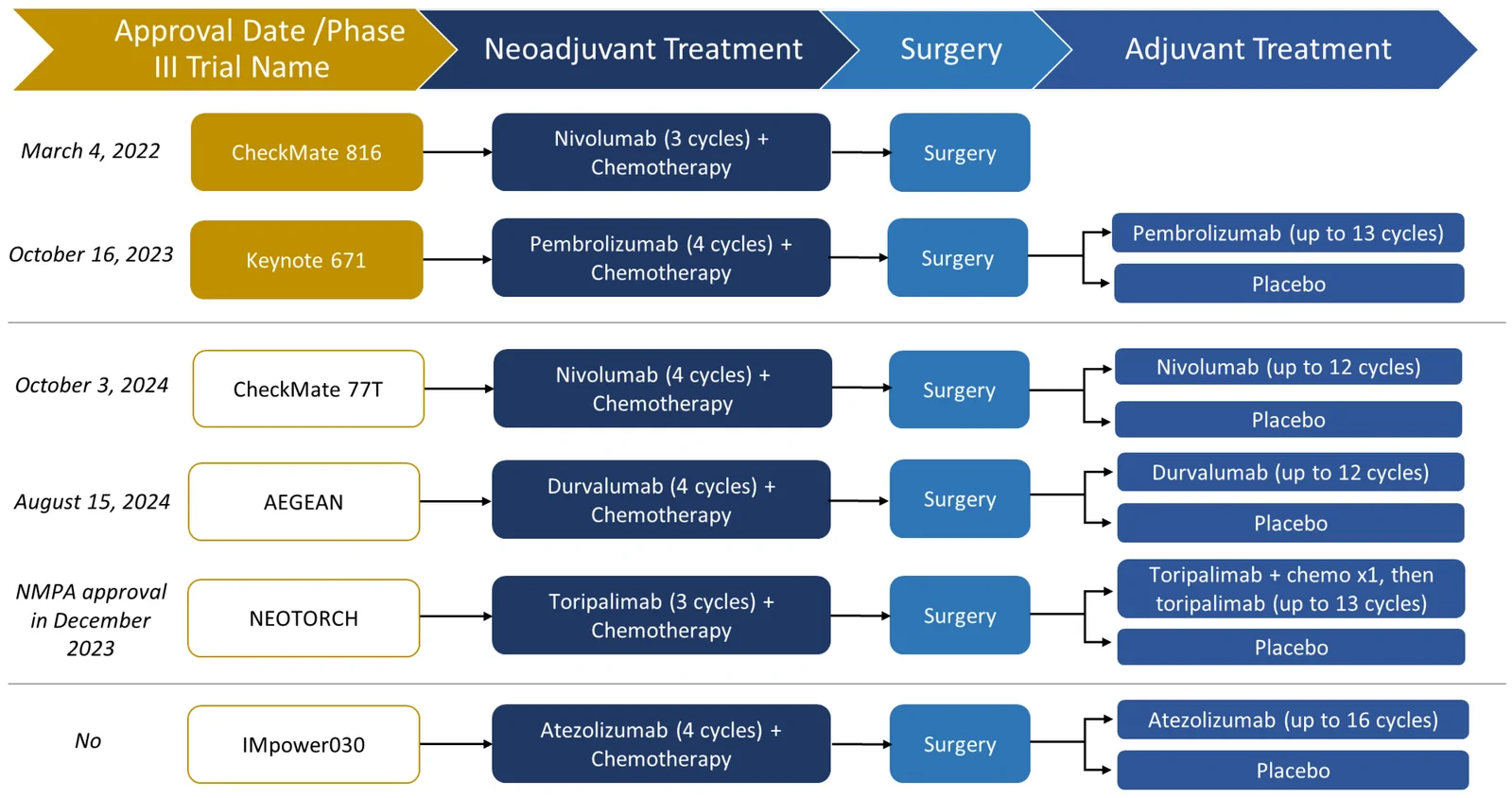
Summary of phase III studies for resectable NSCLC without AGAs. Overview of key design and efficacy outcomes from major phase III trials evaluating perioperative or neoadjuvant chemoimmunotherapy in resectable NSCLC without AGAs. Abbreviations: AGA, actionable genomic alteration; EFS, event-free survival; HR, hazard ratio; ITT, intent-to-treat; MPR, major pathologic response; N2, node-positive (ipsilateral mediastinal); NSCLC, non-small cell lung cancer; OS, overall survival; pCR, pathologic complete response; PD-L1, programmed death-ligand 1.

Figure 2 summarizes the available neoadjuvant/perioperative chemoimmunotherapy regimens in North America, with detailed trial information summarized in Table 1. 

## 3. Perioperative Targeted Therapies for NSCLC with AGAs

Approximately 35% of patients with NSCLC harbor AGAs, with the prevalence approaching 50% among those with nonsquamous histology [52,53]. The LEADER trial found AGAs in 35% of patients with resectable stage IA2–III NSCLC [54]. The identification of these alterations, particularly *EGFR* mutations, *ALK* rearrangements, and *RET* fusions, is increasingly informing curative-intent treatment strategies. This molecular distinction is clinically important because oncogene-driven tumors generally derive less benefit from ICIs than tumors without AGAs, and NSCLC patients with these AGAs were excluded from or underrepresented in several pivotal perioperative immunotherapy trials; although KEYNOTE-671 permitted enrollment of patients with *EGFR* or *ALK* alterations, the resulting molecularly defined subgroups were too small to support meaningful conclusions. Accordingly, obtaining adequate tissue for comprehensive molecular profiling before initiation of perioperative systemic therapy is increasingly important to identify actionable alterations and avoid exposing patients with AGA tumors to potentially ineffective or unnecessarily toxic immunotherapy.

The limited efficacy of ICI in NSCLC harboring AGAs is attributable to several biological factors, including low tumor mutational burden, reduced neoantigen load, and an immune-cold or immune-excluded tumor microenvironment [55,56,57]. Oncogenic signaling may further promote immune evasion through impaired interferon signaling, reduced antigen presentation, TGF-β-mediated suppression of T-cell activity, and recruitment of immunosuppressive myeloid cells [58,59,60,61]. Consequently, PD-L1 expression alone may not reliably predict immunotherapy benefit in these tumors, and ORRs and PFS with ICIs are generally inferior to those observed in AGA-negative NSCLC [62].

An additional consideration is the potential for increased toxicity when ICI is followed by certain tyrosine kinase inhibitors. Sequential ICI-to-TKI therapy has been associated with pneumonitis rates as high as 38% with osimertinib after durvalumab and 25% with alectinib after prior ICI exposure, as well as grade 3–4 hepatotoxicity in up to 45.5% of patients receiving crizotinib after an ICI [63,64]. The risk appears greatest when the TKI is initiated within three months of the last ICI dose [65].

Together, the limited efficacy of immunotherapy, potential toxicity of ICI-to-TKI sequencing, and dependence of these tumors on a dominant oncogenic pathway provide a strong rationale for incorporating targeted therapy earlier in the treatment course. Recent phase III trials have consequently evaluated genotype-directed therapy across the adjuvant, neoadjuvant, and perioperative settings, with the most mature evidence currently available for *EGFR*-, *ALK*-, and *RET*-driven NSCLC (Table 2).

### 3.1. Adjuvant Targeted Therapy

Adjuvant tyrosine kinase inhibitors (TKIs) have substantially improved DFS in patients with resected *EGFR*-mutant and *ALK*-rearranged NSCLC, with an OS benefit demonstrated for adjuvant osimertinib in classic *EGFR*-mutant NSCLC. In contrast, adjuvant targeted therapies for other AGAs, including *RET* fusions, remain investigational despite promising emerging evidence. The magnitude of benefit in stage IB disease remains less well defined, particularly for *ALK*-rearranged and *RET* fusion-positive NSCLC. Adequate tissue acquisition and comprehensive molecular profiling before treatment initiation are therefore essential to guide optimal perioperative treatment selection.

#### 3.1.1. *EGFR*-Mutant NSCLC: ADAURA

The phase III, double-blind ADAURA trial randomized 682 patients with completely resected stage IB–IIIA (AJCC 7th edition) NSCLC harboring an *EGFR* exon 19 deletion or exon 21 L858R mutation to osimertinib 80 mg daily for 3 years versus placebo. Adjuvant platinum-based chemotherapy was permitted before randomization but was not required; approximately 60% of the overall population and 76% of patients with stage II–IIIA disease received chemotherapy [66]. At the final DFS analysis, osimertinib maintained a substantial benefit in patients with stage II–IIIA disease (HR, 0.23; 95% CI, 0.18–0.30), with 4-year DFS rates of 70% versus 29% [88]. CNS DFS was also improved (HR, 0.24; 95% CI, 0.14–0.42), with fewer local, distant, and CNS recurrences [88]. This improvement in disease control subsequently translated into a statistically significant OS benefit. At 5 years, OS among patients with stage II–IIIA disease was 85% with osimertinib versus 73% with placebo (HR, 0.49; 95.03% CI, 0.33–0.73); in the overall stage IB–IIIA population, the corresponding rates were 88% and 78% (HR, 0.49; 95.03% CI, 0.34–0.70) [67]. In both populations, osimertinib reduced the risk of death by 51%, establishing 3 years of adjuvant osimertinib as a standard treatment after complete resection of eligible *EGFR*-mutant NSCLC [67]. An exploratory 8-year landmark analysis presented at the 2026 WCLC demonstrated sustained OS benefit after completion of adjuvant therapy. Eight-year OS was 74% with osimertinib versus 58% with placebo among patients with stage II–IIIA disease (HR, 0.53; 95% CI, 0.38–0.75) and 79% versus 64% in the overall stage IB–IIIA population (HR, 0.52; 95% CI, 0.39–0.71) [68].

ADAURA is notable because its substantial reduction in recurrence translated into an OS benefit, unlike earlier adjuvant *EGFR*-TKI studies that primarily delayed relapse. Osimertinib also reduced distant and CNS recurrence, suggesting an effect on both the timing and pattern of relapse, despite greater use of subsequent *EGFR*-directed therapy in the placebo group. However, chemotherapy use was not randomized, stage-specific analyses were exploratory, and the absolute benefit in stage IB disease was more modest. The trial was also limited to *EGFR* exon 19 deletion and L858R mutations in patients with good performance status and did not define the optimal treatment duration or role of molecular residual disease. Lastly, the optimal duration of therapy remains uncertain.

#### 3.1.2. *ALK*-Positive NSCLC: ALINA 

Extending the precision adjuvant approach to *ALK*-rearranged disease, the phase III ALINA trial randomized 257 patients with completely resected stage IB-IIIA *ALK*-positive NSCLC to alectinib 600 mg twice daily for 2 years versus four cycles of platinum-based chemotherapy. Unlike ADAURA, ALINA directly compared targeted therapy with chemotherapy rather than permitting chemotherapy before randomization. 

At the initial primary analysis, alectinib reduced the risk of recurrence or death by 76% among patients with stage II–IIIA disease, with 2-year DFS rates of 93.8% versus 63.0% with chemotherapy [69]. Similar benefit was observed in the overall stage IB–IIIA population and across prespecified subgroups. Alectinib also reduced the risk of CNS recurrence or death by 78%, consistent with its established intracranial activity in advanced *ALK*-positive NSCLC [69]. The benefit remained durable with longer follow-up, with results presented at the European Society for Medical Oncology 2025 Congress. With approximately 4 years of follow-up, DFS in the overall population was 75.5% with alectinib versus 47.0% with chemotherapy, corresponding to a 65% reduction in the risk of recurrence or death [70]. Four-year CNS DFS rates were 90.4% and 76.1%, respectively [70]. OS continued to favor alectinib numerically, with 4-year rates of 98.4% versus 92.4%, although OS remains immature [70]. Notably, 81.7% of patients who experienced recurrence after chemotherapy subsequently received an ALK inhibitor, which may attenuate an eventual OS difference. A 2026 exploratory analysis further demonstrated consistent DFS benefit across disease stages when reclassified according to the AJCC 8th edition, as well as across nodal status and tumor-size subgroups [71].

ALINA, therefore, established 2 years of adjuvant alectinib as a chemotherapy-sparing standard after complete resection of eligible *ALK*-positive NSCLC. However, because the trial compared alectinib with chemotherapy rather than evaluating sequential chemotherapy followed by alectinib, it did not determine whether chemotherapy provides additional benefit when combined with adjuvant ALK inhibition. The optimal treatment duration remains uncertain, particularly because additional recurrences occurred after completion of the 2-year course. 

#### 3.1.3. *ALK*-Positive NSCLC: ELEVATE

Complementing the findings of ALINA, the phase III ELEVATE trial evaluated the efficacy of adjuvant ensartinib following complete resection of *ALK*-positive NSCLC after stage-appropriate chemotherapy. The trial randomized 274 patients with completely resected stage IB–IIIB *ALK*-positive NSCLC to ensartinib 225 mg daily or placebo for up to 24 months. Adjuvant chemotherapy was required for patients with stage IIB–IIIB disease unless declined or not tolerated, whereas its use in stage IB–IIA disease was at the treating physician’s discretion. Overall, approximately 70% of patients received adjuvant chemotherapy, including 89.1% of those with stage IIB–IIIB disease and 23.5% of those with stage IB–IIA disease. The primary analysis demonstrated a substantial DFS benefit. Among patients with stage II–IIIB disease, ensartinib reduced the risk of recurrence or death by 80%, with 24-month DFS rates of 86.4% versus 53.5% with placebo [72]. Similar benefit was observed in the overall stage IB–IIIB population, in which the corresponding rates were 87.3% and 57.2% [72]. Ensartinib also reduced the risk of CNS recurrence or death by 78%; brain recurrence occurred in 3.6% of patients receiving ensartinib compared with 12.4% receiving placebo [72]. OS remained immature, with only three deaths reported at the interim analysis.

Together, ALINA and ELEVATE support postoperative *ALK* inhibition but address distinct treatment strategies. ALINA demonstrated that alectinib could replace adjuvant chemotherapy, whereas ELEVATE (more closely resembling the ADAURA design) demonstrated benefit from ensartinib after chemotherapy when indicated. ELEVATE did not directly compare ensartinib with alectinib nor establish that chemotherapy is necessary before adjuvant *ALK* inhibition. Interpretation is also limited by the exclusively Chinese study population, relatively short follow-up, immature OS data, and few events among patients with stage IB disease. Ensartinib is FDA-approved for *ALK*-positive locally advanced or metastatic NSCLC but does not have FDA approval for adjuvant treatment of resected *ALK*-positive NSCLC.

Both alectinib and ensartinib are second-generation *ALK* TKIs. In contrast, the phase III ALCHEMIST-ALK (E4512) trial demonstrated that the first-generation ALK inhibitor crizotinib for up to 2 years did not improve DFS compared with observation/placebo, suggesting that efficacy in advanced disease cannot necessarily be extrapolated across ALK inhibitors to the curative-intent setting [89]. The third-generation ALK inhibitor lorlatinib has demonstrated superior PFS and intracranial disease control compared with crizotinib in advanced *ALK*-positive NSCLC, with the 7-year CROWN analysis demonstrating median PFS still not reached and no new intracranial progression events after the first 30 months of therapy [90]; however, its efficacy as adjuvant therapy has not been established. Whether its greater potency and central nervous system (CNS) activity could translate into improved survival outcomes must be balanced against its distinctive toxicity profile, including hyperlipidemia, neurocognitive and mood effects, and weight gain. Further studies are needed to determine whether more potent next-generation ALK inhibitors can improve upon the substantial DFS benefit already achieved with adjuvant alectinib.

#### 3.1.4. RET Fusion-Positive NSCLC: LIBRETTO-432

Extending the adjuvant targeted-therapy paradigm to *RET* fusion-positive disease, the phase III, LIBRETTO-432 trial randomized 151 patients with stage IB–IIIA NSCLC to selpercatinib or placebo for up to 3 years after definitive curative-intent therapy. Selpercatinib was administered at 160 mg twice daily for patients weighing at least 50 kg and 120 mg twice daily for those weighing less than 50 kg. Nearly all patients underwent surgery, although definitive radiotherapy was permitted, and adjuvant systemic therapy was administered when indicated. Among patients with stage II–IIIA disease, more than 90% had received adjuvant chemotherapy before randomization, making the trial design more analogous to ADAURA and ELEVATE than to ALINA.

The primary analysis demonstrated a marked EFS benefit. Among patients with stage II–IIIA disease, 2-year EFS was 92% with selpercatinib versus 61% with placebo, corresponding to an 83% reduction in the risk of recurrence, progression, or death [73]. In the overall stage IB–IIIA population, the corresponding rates were 94% and 70% [73]. Results from blinded independent central review were consistent with the investigator-assessed analysis. OS remained immature, with only three deaths reported, all from disease progression in the placebo group; patients assigned to placebo were also permitted to cross over to selpercatinib after recurrence.

LIBRETTO-432 provides the first randomized phase III evidence supporting adjuvant RET inhibition after definitive local treatment. The magnitude of benefit was similar to that observed with osimertinib in ADAURA and alectinib in ALINA, although cross-trial comparisons should be interpreted cautiously. Important uncertainties include the small study population, limited follow-up, and immature OS data. More than half of the patients also remained on assigned therapy at the data cutoff, limiting assessment of the durability of benefit after treatment completion. Selpercatinib is currently approved in the U.S. for advanced or metastatic *RET* fusion-positive NSCLC but does not have a specific adjuvant indication.

Although several pivotal neoadjuvant and adjuvant trials enrolled patients with stage IB NSCLC, these patients generally represented a small proportion of the study populations. In particular, the stage IB cohorts in ALINA, LIBRETTO-432, and ELEVATE were limited, restricting the statistical power to establish stage-specific treatment benefits. Consequently, the overall efficacy results should not be assumed to apply equally to stage IB disease. Treatment decisions for stage IB NSCLC should consider tumor size, high-risk pathological features, actionable genomic alterations, and the strength of evidence supporting each treatment strategy. Further prospective studies with adequate representation of stage I patients are warranted.

### 3.2. Neoadjuvant and Perioperative Targeted Therapy

Although adjuvant *EGFR*- and *ALK*-directed therapies have demonstrated substantial clinical benefits, the efficacy, optimal sequencing, and duration of neoadjuvant and perioperative targeted therapies for resectable NSCLC harboring AGAs remain under investigation.

#### 3.2.1. *EGFR*-Mutant NSCLC: NeoADAURA

NeoADAURA provides the strongest phase III evidence to date for neoadjuvant targeted therapy in resectable *EGFR*-mutated NSCLC [80]. The trial randomized 358 patients with resectable stage II–IIIB disease to neoadjuvant osimertinib plus platinum-based chemotherapy, osimertinib monotherapy, or placebo plus platinum-based chemotherapy. Treatment was administered for at least 9 weeks before surgery, and postoperative osimertinib was permitted. MPR occurred in 26% of patients receiving osimertinib plus chemotherapy, 25% receiving osimertinib alone, and 2% receiving chemotherapy alone. pCR rates were 4%, 9%, and 0%, respectively. Among patients with baseline N2 disease, nodal downstaging occurred in 53% with either osimertinib regimen versus 21% with chemotherapy alone. Surgical resection and R0 resection rates remained high across groups, and progressive disease prevented surgery less often with osimertinib-containing therapy. Notably, osimertinib monotherapy produced an MPR rate similar to that observed with osimertinib plus chemotherapy (25% vs. 26%), while grade 3 or higher adverse events occurred less frequently with osimertinib alone (13% vs. 36%). However, the trial was not designed to compare the two osimertinib-containing regimens directly, and these results therefore do not establish equivalence or exclude an incremental benefit from adding chemotherapy.

EFS remains immature, and more than 80% of patients in each group subsequently received adjuvant osimertinib. Longer-term outcomes will therefore reflect an integrated perioperative strategy rather than isolate the effect of preoperative treatment. Moreover, MPR has not been validated as a surrogate for survival in *EGFR*-mutant NSCLC treated with TKIs. NeoADAURA establishes clear preoperative activity and surgical feasibility, but its practice-changing significance will depend on whether this early pathologic benefit translates into durable EFS and whether chemotherapy adds meaningful benefit beyond osimertinib alone.

#### 3.2.2. Promising Neoadjuvant Targeted Therapies

Recent phase II studies have demonstrated encouraging pathological responses and surgical feasibility with neoadjuvant ALK and KRAS G12C inhibitors.

*ALK*-rearranged NSCLC. The phase II NAUTIKA1 trial (NCT04302025) evaluated eight weeks of neoadjuvant alectinib (600 mg twice daily) followed by surgery, optional platinum-based chemotherapy, and up to two years of adjuvant alectinib in patients with resectable stage IB–IIIB *ALK*-positive NSCLC. In the primary analysis presented at the 2026 World Conference on Lung Cancer (WCLC), 48 patients were enrolled. Among 43 pathologically evaluable patients, MPR and pCR rates were 55.8% (24/43) and 18.6% (8/43), respectively. The radiographic ORR was 60%, and R0 resection was achieved in 95% of patients undergoing resection. At a median follow-up of 20.8 months, the estimated 2-year EFS, disease-free survival (DFS), and OS rates were 94%, 96%, and 97%, respectively. These findings support the feasibility and promising activity of neoadjuvant ALK inhibition, although its incremental benefit over established adjuvant alectinib remains uncertain.

KRAS G12C-mutant NSCLC. Updated results from the KRAS G12C cohort of NAUTIKA1, also presented at WCLC 2026, demonstrated encouraging activity with neoadjuvant divarasib, a selective KRAS G12C inhibitor. Twenty patients with resectable KRAS G12C-mutant NSCLC received eight weeks of divarasib before surgery. Among 19 pathologically evaluable patients, MPR and pCR rates were 42% (8/19) and 16% (3/19), respectively, with a radiographic ORR of 55% (11/20). All 19 patients who underwent surgery achieved R0 resection, without reported surgical delays or intraoperative complications. These findings provide early proof of concept for neoadjuvant KRAS G12C inhibition, although the small sample size and lack of randomized comparisons limit conclusions regarding long-term efficacy. 

Collectively, these findings highlight the potential of biomarker-directed neoadjuvant therapy in resectable NSCLC. However, neoadjuvant ALK and KRAS G12C inhibition remain investigational, and larger randomized studies with mature survival outcomes are needed to establish their roles in routine clinical practice. 

## 4. Clinical Application 

**Figure 3:**
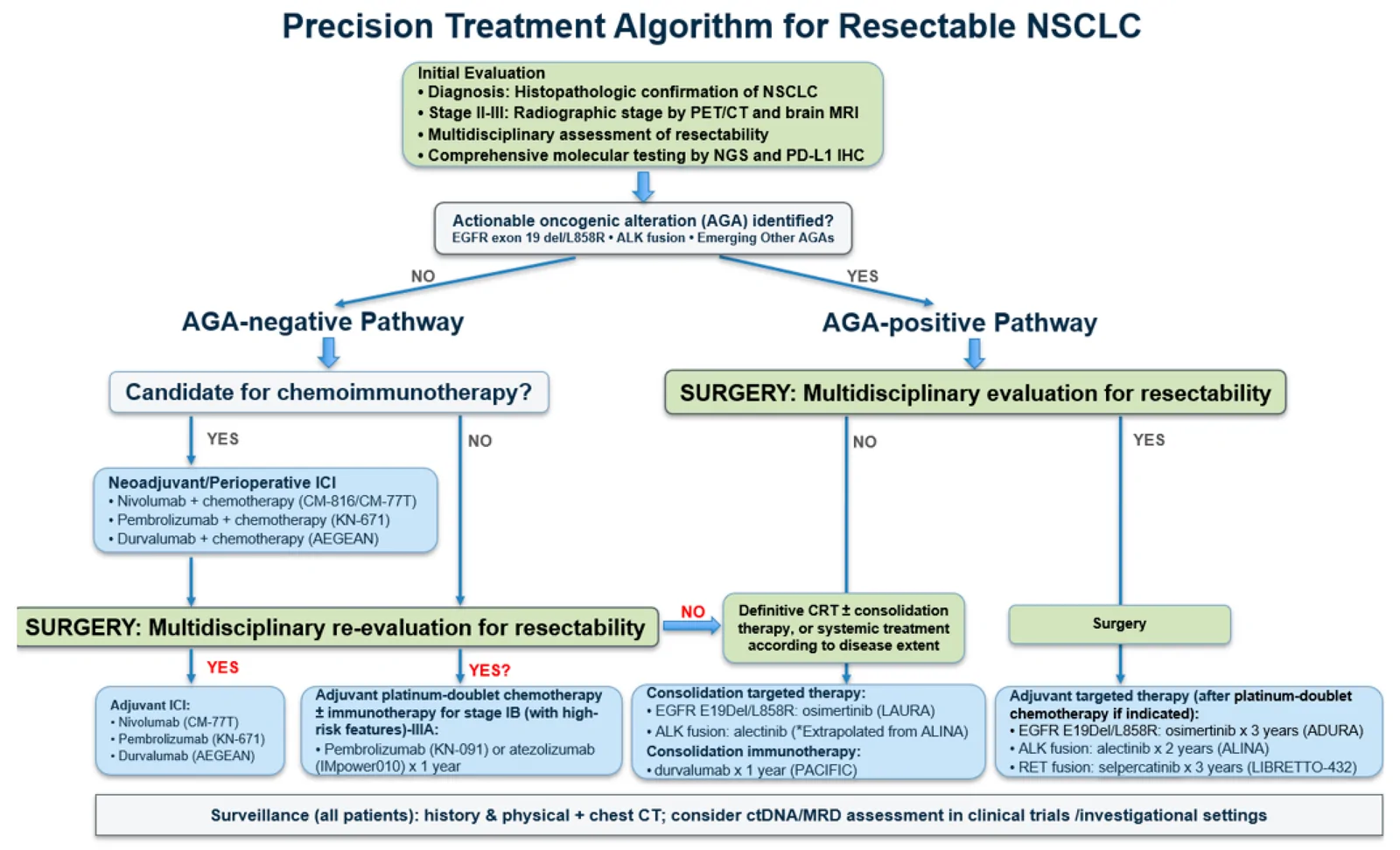
Precision treatment algorithm for resectable NSCLC. Following histopathologic confirmation and radiographic staging, multidisciplinary assessment of resectability and comprehensive biomarker testing, including next-generation sequencing (NGS) and PD-L1 immunohistochemistry, should be performed before treatment selection. Patients with actionable genomic alterations (AGAs) are directed toward surgery followed by genotype-specific strategies, including adjuvant targeted therapy for *EGFR* exon 19 deletion/L858R mutations and *ALK* fusions, with emerging targeted approaches for other oncogenic drivers. Patients without AGAs who are candidates for chemoimmunotherapy may receive neoadjuvant or perioperative platinum-based chemotherapy plus an immune checkpoint inhibitor (ICI), followed by surgery, with postoperative ICI according to the selected perioperative regimen. Patients undergoing upfront surgery may receive adjuvant platinum-doublet chemotherapy with or without immunotherapy according to pathologic stage, biomarker status, and clinical risk. Patients deemed unresectable after multidisciplinary evaluation may receive definitive concurrent chemoradiotherapy (CRT), followed by appropriate consolidation therapy according to molecular status and available evidence. For those patients who progressed to metastatic disease, they would be treated as having metastatic disease. Post-treatment surveillance includes clinical assessment and chest CT, while circulating tumor DNA-based molecular residual disease (ctDNA-MRD) assessment remains investigational and may help guide future response-adapted treatment strategies. * Although adjuvant alectinib has demonstrated significant disease-free survival benefit in resected *ALK*-positive NSCLC in the phase III ALINA trial, its use as consolidation therapy following definitive CRT for unresectable stage III *ALK*-positive NSCLC represents an extrapolation from the adjuvant setting. This approach remains investigational, without prospective phase III randomized evidence establishing its efficacy. Abbreviations: AGA, actionable genomic alteration; CT, computed tomography; ctDNA, circulating tumor DNA; ICI, immune checkpoint inhibitor; MRD, molecular residual disease; NGS, next-generation sequencing; NSCLC, non-small cell lung cancer; PD-L1, programmed death-ligand 1.

Successful implementation of precision perioperative therapy requires a practice change in close multidisciplinary collaboration among medical oncologists, thoracic surgeons, radiation oncologists, pathologists, radiologists, and molecular diagnostic teams. The integration of clinical evaluation for stage, resectability, molecular profile, and treatment selection provides a practical framework for selecting among contemporary perioperative strategies for resectable NSCLC (Figure 3); such an approach would require several changes in the diagnostic workups, as detailed below. 

### 4.1. Mediastinal Staging and Stage Migration

Mediastinal staging has evolved toward minimally invasive endoscopic techniques, particularly endobronchial ultrasound-guided transbronchial needle aspiration (EBUS-TBNA), with or without endoscopic ultrasound (EUS) [91,92]. These approaches provide comprehensive sampling of mediastinal and hilar lymph nodes with lower morbidity than surgical staging. Consequently, routine confirmatory mediastinoscopy can generally be omitted after adequate negative endoscopic staging and is increasingly reserved for patients with persistent clinical or radiographic suspicion or nodal stations inaccessible by EBUS/EUS [92].

The 9th edition of the TNM classification for lung cancer came into effect in January 2025, while most contemporary perioperative trials were conducted using the 7th or 8th editions [93,94]. An important change is the subdivision of N2 disease into N2a (single-station mediastinal involvement) and N2b (multiple-station involvement), reflecting their distinct prognoses [93]. The 9th edition also reclassifies T1N1 disease from stage IIB to IIA and introduces additional refinements intended to improve prognostic stratification [93]. These changes should be considered when translating results from earlier perioperative trials into contemporary clinical practice.

### 4.2. Limited Evidence for Neoadjuvant, Perioperative, and Adjuvant Immunotherapy in Stage I NSCLC

The role of neoadjuvant, perioperative, and adjuvant ICIs in stage I NSCLC without AGAs remains incompletely defined. Although CheckMate-816 enrolled patients with stage IB (tumors ≥4 cm)–IIIA NSCLC according to AJCC 7th edition staging, only approximately 5% had stage IB disease, limiting the reliability of stage-specific efficacy estimates. Importantly, tumors measuring > 4–5 cm classified as stage IB under AJCC 7th edition are classified as stage IIA under AJCC 8th edition. Other pivotal neoadjuvant or perioperative trials, including CheckMate-77T, KEYNOTE-671, AEGEAN, and NADIM II, did not enroll patients with contemporary stage I disease. Consequently, randomized evidence supporting neoadjuvant or perioperative chemoimmunotherapy for AJCC 8th edition stage I NSCLC is lacking.

In the adjuvant setting, PEARLS/KEYNOTE-091 and IMpower010 enrolled patients with completely resected stage IB tumors ≥ 4 cm under AJCC 7th edition staging. KEYNOTE-091 demonstrated improved DFS with pembrolizumab in the overall study population (HR, 0.76; 95% CI, 0.63–0.91), leading to FDA approval in January 2023 for stage IB (≥4 cm)–IIIA NSCLC following resection and platinum-based chemotherapy. However, the stage IB subgroup was relatively small, and definitive stage-specific benefit has not been established. Similarly, IMpower010 demonstrated improved DFS with adjuvant atezolizumab, particularly in patients with stage II–IIIA disease and PD-L1 expression ≥ 1%. At the final DFS analysis after at least five years of follow-up, the DFS HR in the overall stage IB–IIIA intention-to-treat population was 0.85 (95% CI, 0.71–1.01), whereas the stage II–IIIA PD-L1 ≥1% population maintained a DFS benefit (HR, 0.70; 95% CI, 0.55–0.91). The FDA-approved indication for adjuvant atezolizumab is restricted to stage II–IIIA NSCLC with PD-L1 expression ≥1%.

Collectively, these findings highlight the limited direct evidence supporting perioperative or adjuvant immunotherapy for contemporary AJCC 8th edition stage I NSCLC, particularly tumors <4 cm without nodal involvement. Upfront surgical resection remains the preferred treatment for medically operable stage I disease, with postoperative systemic therapy considered according to pathological findings and established indications. Further prospective studies are warranted to identify high-risk stage I populations that may benefit from immunotherapy.

### 4.3. Surgical Decision-Making and Surgical Outcomes

Surgical management of resectable NSCLC requires multidisciplinary assessment of anatomic resectability, mediastinal nodal involvement, cardiopulmonary fitness, and the likelihood of achieving an R0 resection [95,96]. Although selected patients with non-bulky, single-station N2 disease may benefit from multimodality treatment incorporating surgery, multi-station N2 disease and bulky or fixed mediastinal adenopathy require careful individualized evaluation [95,97]. Following neoadjuvant therapy, restaging should assess disease progression, nodal response, operative fitness, and continued feasibility of complete resection. Surgical planning should prioritize oncologic clearance while preserving lung function when feasible, with lobectomy generally preferred when appropriate. Sleeve resection and pneumonectomy may be considered for selected central tumors in appropriate surgical candidates [98]. Minimally invasive approaches may be feasible in selected patients; however, treatment-related fibrosis, inflammation, and altered tissue planes can complicate hilar dissection and necessitate conversion to thoracotomy or more extensive resection [17,97,98].

After neoadjuvant chemoimmunotherapy, multidisciplinary reassessment for surgery should integrate interval imaging, the clinical course, treatment toxicity, and reassessment. Response may facilitate resection in selected patients, whereas progression or an unfavorable risk–benefit assessment may redirect treatment toward definitive chemoradiation. PET findings and ctDNA clearance are potentially informative but cannot independently establish pathologic nodal sterilization or justify omission of surgery. The reassessment is particularly important for stage III NSCLC patients with borderline resectability before neoadjuvant treatment. The phase II MDT-BRIDGE trial (NCT05925530) evaluated a multidisciplinary team (MDT)-guided treatment strategy in patients with resectable or borderline-resectable stage IIB–IIIB (N2), *EGFR*/*ALK* wild-type NSCLC [99,100]. Following two cycles of neoadjuvant durvalumab plus platinum-based chemotherapy, patients underwent MDT reassessment of resectability. Those deemed resectable received an additional one to two cycles of chemoimmunotherapy followed by surgery and adjuvant durvalumab, whereas patients deemed unresectable received definitive chemoradiotherapy followed by consolidation durvalumab. In the primary analysis presented at WCLC 2026 [100], 142 patients were treated, including 50 (35.2%) considered borderline resectable at baseline. Overall, 106 patients (74.6%) underwent surgical resection, with an R0 resection rate of 96.2%. Among initially borderline-resectable patients, 76% were considered resectable at MDT reassessment, and 62% ultimately underwent surgery. An additional 25 patients (17.6%) received definitive chemoradiotherapy, resulting in 92.3% of patients receiving curative-intent local therapy. The pCR rate was 23.2% in the overall population and 27.3% among patients considered resectable at MDT reassessment. The estimated 12-month EFS rate was 88.5%. These findings support the feasibility of an MDT-guided, response-adapted treatment strategy that integrates surgery and definitive chemoradiotherapy to preserve curative-intent treatment options. However, the nonrandomized design and immature survival data limit conclusions regarding comparative efficacy. 

Surgical outcomes provide an important complement to pathological response and EFS when evaluating perioperative treatment strategies. A recent systematic review and meta-analysis of 27 trials involving 2691 patients reported pooled rates of 3% for intraoperative complications, 27% for postoperative complications, and 1% for postoperative mortality; minimally invasive surgery was used in 47% of resections, with a 20% conversion rate and surgical delays in 9% of cases [101]. These findings underscore the importance of evaluating perioperative safety alongside oncologic outcomes. Disease progression, treatment-related toxicity, declining functional status, and persistent unresectability may delay or prevent surgery in some patients [17,102]. Integrating surgical expertise into treatment selection and post-treatment reassessment is therefore essential to optimize the balance between systemic disease control, complete resection, and perioperative safety [95,96,97]. Table 3 summarizes these surgical outcomes based on the available data. 

### 4.4. Biomarker Testing for Resectable NSCLC

As perioperative treatment options continue to expand, adequate tissue acquisition and timely biomarker testing before treatment initiation have become increasingly important. The 2026 NCCN, ESMO, and IASLC recommendations emphasize early assessment of actionable oncogenic drivers, particularly *EGFR* and *ALK*, together with PD-L1 expression to guide selection between targeted and immunotherapy-based perioperative strategies (Figure 3) [95,103,104]. The CAP/IASLC/AMP molecular-testing guideline also supports molecular testing in early-stage lung adenocarcinoma and the use of multiplexed testing approaches, although its currently published recommendations predate many recent advances in adjuvant and neoadjuvant targeted therapy [105]. As of 2026, the VA Oncology Lung Cancer Clinical Pathway takes a comprehensive approach, recommending PD-L1 TPS testing for all NSCLC and DNA- and RNA-based comprehensive genomic profiling for nonsquamous tumors in the relevant resectable-disease setting [106]. Collectively, these recommendations underscore the importance of obtaining adequate diagnostic tissue and completing clinically relevant biomarker testing before initiation of neoadjuvant or perioperative systemic therapy.

### 4.5. Neoadjuvant Versus Adjuvant ICIs

No randomized trial has directly compared neoadjuvant with adjuvant-only immunotherapy in resectable NSCLC. However, neoadjuvant and perioperative chemoimmunotherapy have consistently improved EFS in several phase III trials, with statistically significant OS benefits now demonstrated in CheckMate-816 and KEYNOTE-671 [18,29]. In contrast, adjuvant-only immunotherapy has shown less consistent efficacy, with IMpower010 and PEARLS/KEYNOTE-091 demonstrating improved DFS without a significant OS benefit to date [26]. Although indirect comparisons across trials cannot establish the superiority of one treatment sequence over another, these findings support the incorporation of immunotherapy before surgery in appropriately selected patients. 

The optimal sequencing of immunotherapy remains an important unanswered question. The pragmatic phase III PROSPECT-Lung trial (Alliance A082304/SWOG S2402; NCT06632327) was designed to directly compare perioperative chemoimmunotherapy with surgery followed by adjuvant chemoimmunotherapy but closed in August 2026 due to poor accrual. Meanwhile, two randomized phase III trials, SWOG S2414/INSIGHT (NCT06498635) and ETOP 25-23/ADOPT-lung (NCT06284317), are investigating the benefit of adjuvant durvalumab versus observation following neoadjuvant chemoimmunotherapy and surgical resection. SWOG S2414/INSIGHT (NCT06498635) is evaluating adjuvant durvalumab versus surveillance in patients achieving a pCR after neoadjuvant chemoimmunotherapy and surgery. INSIGHT specifically enrolls patients achieving pCR, whereas ADOPT-lung includes patients with and without pCR. These trials may help define the incremental benefit of postoperative immunotherapy and inform response-adapted treatment strategies, including potential treatment de-escalation in patients achieving pCR. The ECOG-ACRIN CLEAR trial (NCT06732401), which has not yet opened to accrual, is designed to evaluate the addition of ceralasertib to adjuvant durvalumab in patients without pCR. Together, these studies highlight the growing interest in response-adapted postoperative treatment strategies. Integration of pathological response, including pCR and MPR, with ctDNA-defined MRD may ultimately help identify patients who benefit from additional postoperative therapy and those in whom treatment can be safely omitted. 

### 4.6. Neoadjuvant Versus Perioperative ICIs

There is growing interest in optimizing the duration and sequencing of ICIs to maximize clinical benefit while minimizing unnecessary treatment and toxicity in patients with resectable NSCLC. Figure 2 summarizes the pivotal phase III trials that have shaped the evolving treatment paradigm. Unlike CheckMate-816, which evaluated neoadjuvant nivolumab plus chemotherapy without adjuvant immunotherapy, CheckMate-77T incorporated adjuvant nivolumab following neoadjuvant nivolumab plus platinum-based chemotherapy and surgical resection. Exploratory analyses of CheckMate-77T suggested favorable event-free survival (EFS) with the perioperative regimen, including among patients achieving a pCR. However, these findings should be interpreted cautiously because CheckMate-77T evaluated the entire perioperative strategy rather than isolating the incremental benefit of adjuvant nivolumab. Furthermore, cross-trial comparisons cannot establish whether postoperative ICIs provide additional benefit following neoadjuvant chemoimmunotherapy. Thus, the optimal role and duration of adjuvant ICIs remain uncertain, particularly in patients achieving deep pathological responses.

To date, no completed randomized trial has directly compared neoadjuvant chemoimmunotherapy alone with the same neoadjuvant regimen followed by adjuvant ICI therapy. Consequently, the incremental clinical benefit of postoperative ICIs remains uncertain. Notably, as of October 2026, the VA Oncology Lung Cancer Clinical Pathway (April 2026 version) recommends neoadjuvant platinum-based chemotherapy plus nivolumab followed by surgery and surveillance after complete resection, without routine continuation of postoperative ICIs as part of a perioperative chemoimmunotherapy regimen [106]. Prospective studies incorporating pathologic response and ctDNA-defined MRD may help identify patients who benefit from continued postoperative immunotherapy and those in whom treatment can be safely omitted.

### 4.7. Selecting an ICI for Neoadjuvant Therapy

Nivolumab was the first ICI to receive FDA approval for neoadjuvant treatment in March 2022 following the results of CheckMate-816, and subsequently received perioperative FDA approval in October 2024 based on CheckMate 77T. In October 2023, pembrolizumab became the second ICI to receive FDA approval for use in the perioperative setting in combination with platinum-based chemotherapy following improved EFS and pCR rates in KEYNOTE-671. Durvalumab became the third ICI approved in August 2024 for perioperative use based on the AEGEAN study. In contrast, the final analysis of IMpower030 showed numerically improved EFS with perioperative atezolizumab but did not meet the prespecified threshold for statistical significance. NEOTORCH has also completed its final analysis, with perioperative toripalimab plus chemotherapy meeting the prespecified EFS and MPR efficacy boundaries in the stage II–III population, with detailed final results pending. These findings have expanded the evidence base for perioperative chemoimmunotherapy and continue to refine curative-intent treatment strategies for resectable NSCLC. These phase III studies provide level I evidence supporting perioperative chemoimmunotherapy as a standard-of-care approach for patients with resectable NSCLC. However, the optimal regimen remains uncertain, particularly as additional ICIs are approved in combination with neoadjuvant chemotherapy. In the absence of head-to-head comparative trials, regimen selection will remain challenging and will likely be guided by differences in toxicity profiles, treatment schedules and duration, insurance coverage and cost considerations, patient characteristics, and physician experience.

## 5. Translational Insights and Unmet Needs

### 5.1. Tissue Biomarkers

Tissue- and blood-based immune biomarkers associated with clinical response to ICIs and chemoimmunotherapy in advanced or metastatic NSCLC have been evaluated in patients receiving neoadjuvant and perioperative chemoimmunotherapy. Candidate pre-treatment tumor biomarkers include programmed death-ligand 1 (PD-L1) expression by immunohistochemistry, deficient mismatch repair (dMMR), microsatellite instability-high (MSI-H) status, and high tumor mutational burden (TMB) [107]. Among these, PD-L1 tumor proportion score (TPS) expression has been most extensively evaluated in resectable NSCLC. Subgroup analyses from CheckMate-816, KEYNOTE-671, and AEGEAN generally demonstrated greater EFS benefit with nivolumab, pembrolizumab, and durvalumab, respectively, among patients with PD-L1 expression ≥ 1%, whereas the magnitude of EFS benefit was less apparent in patients with PD-L1 expression < 1%. Importantly, however, higher pCR rates with neoadjuvant nivolumab plus chemotherapy in CheckMate-816 were observed regardless of PD-L1 status. Similarly, in AEGEAN, pCR favored durvalumab plus chemotherapy across PD-L1 expression subgroups, although the magnitude of benefit was greatest among patients with PD-L1 expression ≥50%. Thus, PD-L1 expression alone is insufficient for selecting patients for perioperative chemoimmunotherapy, and patients with PD-L1 expression < 1% may still derive benefit from neoadjuvant ICI combined with platinum-based chemotherapy. 

Although dMMR, MSI-H status, and high TMB have been associated with sensitivity to ICI in selected advanced malignancies, their predictive value for neoadjuvant chemoimmunotherapy in NSCLC remains uncertain. MSI-H/dMMR tumors are rare in NSCLC and have not been adequately evaluated in prospective perioperative trials. Similarly, TMB has not consistently predicted pathological or survival benefit from neoadjuvant chemoimmunotherapy; in CheckMate-816, benefit with nivolumab plus chemotherapy was observed irrespective of TMB [107,108]. Additional tissue- and immune-based biomarkers within the tumor microenvironment (TME) are emerging and are discussed in the subsequent section on mechanisms of resistance.

### 5.2. Resistance Mechanisms

Due to the relatively low tumor burden in early-stage NSCLC, tissue-based assessment of surgical specimens remains the gold standard for evaluating pathological treatment response and investigating biomarkers of resistance. This is particularly important following neoadjuvant therapy, when residual viable tumor cells may be sparse and difficult to distinguish within treatment-related fibrosis and other histopathologic changes. A major unmet need in resectable NSCLC is to better characterize mechanisms of resistance to neoadjuvant chemoimmunotherapy, particularly among patients with residual viable tumor after treatment. Current FDA-approved perioperative regimens generally continue the same PD-(L)1 inhibitor after surgery, but whether this strategy is optimal for patients with substantial residual disease or other evidence of treatment resistance remains uncertain. Interactions among residual tumor cells, immune cell populations within the tumor microenvironment (TME), and tertiary lymphoid structures (TLSs) may provide important insights into mechanisms of therapeutic resistance. Defining these mechanisms could help identify patients at increased risk of recurrence, inform individualized postoperative treatment strategies, and support the development of novel approaches to overcome resistance.

Several emerging technologies may facilitate these translational investigations. Multiplex immunohistochemistry and related spatial assays can simultaneously characterize residual tumor cells and immune populations within the TME. Prior studies have demonstrated that increased infiltration by functional cytotoxic CD8+ T cells and reduced regulatory T-cell (Treg) populations are associated with improved response to chemoimmunotherapy [109,110,111]. T-cell receptor (TCR) sequencing provides an additional approach to characterize treatment-associated changes in T-cell clonality and dynamics, with the potential to identify immune signatures associated with response or resistance [5,7,8,112]. Longitudinal quantitative immunophenotyping of peripheral blood mononuclear cells before, during, and after chemoimmunotherapy may similarly identify dynamic immune changes associated with ICI efficacy and toxicity [113,114,115].

Further investigation is needed to validate existing candidate biomarkers and identify novel biomarkers and therapeutic strategies, particularly at the gene-expression and cellular levels from the TME. Among the most transformative approaches in cancer research is the application of single-cell omics technologies. Unlike bulk sequencing, which may obscure intratumoral heterogeneity, single-cell RNA sequencing (scRNA-seq) enables high-resolution characterization of individual tumor, stromal, and immune cell populations. Recent studies of NSCLC treated with neoadjuvant chemoimmunotherapy have identified distinct treatment-associated cellular states and immune populations associated with pathological response, including changes in malignant cells, B cells, T-cell subsets, dendritic cells, monocytes/macrophages, and cancer-associated fibroblasts [116,117,118].

The neoadjuvant setting provides a particularly valuable opportunity for translational investigation because pretreatment biopsy specimens can be compared with post-treatment surgical specimens to characterize therapy-induced changes in the tumor microenvironment and identify residual cellular populations associated with treatment resistance. Integration of single-cell profiling with T-cell receptor sequencing and spatial transcriptomic technologies can further characterize clonal immune responses and define the spatial organization and interactions among residual tumor, stromal, and immune cells, including those within tertiary lymphoid structures [117,119]. Collectively, these approaches may identify molecular pathways and cellular interactions associated with response and resistance to chemoimmunotherapy, potentially enabling improved postoperative risk stratification, identification of novel therapeutic targets, and development of biomarker-guided adjuvant treatment strategies.

### 5.3. Blood-Based Biomarkers: The Role of Circulating Tumor DNA 

Circulating tumor DNA (ctDNA), a component of cell-free DNA shed by tumor cells into the bloodstream, is emerging as a minimally invasive biomarker with potential applications in molecular profiling, treatment monitoring, assessment of therapeutic response, and detection of minimal residual disease (MRD) [120]. In NSCLC, plasma ctDNA testing was initially validated for *EGFR* mutation detection when adequate tumor tissue was unavailable [121]. Beyond genomic profiling, ctDNA has shown promise as a dynamic biomarker of response to neoadjuvant therapy and postoperative recurrence risk. Exploratory analyses from CheckMate-816 and NADIM II demonstrated that ctDNA clearance during neoadjuvant therapy was associated with favorable pathological and clinical outcomes. In CheckMate-816, ctDNA clearance was more frequent with nivolumab plus chemotherapy than with chemotherapy alone and was associated with higher pCR rates, supporting its potential as an early biomarker of treatment efficacy.

Translational analyses from the NADIM study further demonstrated that ctDNA-based MRD assessment may provide prognostic information beyond pathological response in patients with resectable stage III NSCLC treated with perioperative chemoimmunotherapy [21]. MRD positivity following surgery or during adjuvant therapy suggests the persistence of occult micrometastatic disease and was strongly associated with inferior EFS and OS. Several additional NSCLC studies have evaluated ctDNA for detecting and predicting recurrence, although reported sensitivity has varied considerably [121,122,123,124]. 

ctDNA dynamics have also been explored as a means of monitoring immunotherapy response and distinguishing true disease progression from pseudoprogression [96,100,101,102,121,125,126,127]. Serial testing may improve sensitivity for MRD detection compared with assessment at a single time point [103]. In contrast, serial MRD negativity identified a subset of patients without pCR who nevertheless experienced favorable long-term outcomes [21]. These findings suggest that longitudinal MRD assessment may complement pathological response for postoperative risk stratification and could ultimately help identify patients who may benefit from treatment escalation or de-escalation. Despite these promising findings, ctDNA-guided treatment strategies have not yet been established as standard of care in resectable NSCLC. Prospective studies are needed to standardize assay methodology and timing, define clinically actionable thresholds, and determine whether treatment modification based on ctDNA MRD status improves patient outcomes.

### 5.4. Strategies to Enhance the pCR of Neoadjuvant Therapy

Although dual immune checkpoint blockade has demonstrated clinical benefit in advanced or metastatic NSCLC, its role in neoadjuvant and perioperative treatment of resectable disease remains investigational [12,19]. Given that a substantial proportion of patients do not achieve pCR with currently approved chemoimmunotherapy regimens and remain at risk for recurrence, strategies to increase pathological response and improve long-term outcomes are needed.

In an exploratory cohort of CheckMate-816, neoadjuvant nivolumab (3 mg/kg every 2 weeks for three cycles) combined with ipilimumab (1 mg/kg for cycle 1 only) was compared with chemotherapy in patients with resectable NSCLC, with optional adjuvant chemotherapy and/or radiotherapy permitted (Figure 2). The combination was generally well tolerated, with similar rates of definitive surgery between treatment groups. pCR (20.4% vs. 4.6%; OR, 5.14; 95% CI, 1.91–13.80) and MPR (28.3% vs. 14.8%; OR, 2.15; 95% CI, 1.13–4.10) rates were higher with dual immunotherapy than with chemotherapy. Although EFS (54.8 vs. 20.9 months; HR, 0.77; 95% CI, 0.51–1.15) and OS (not reached in either arm; HR, 0.73; 95% CI, 0.47–1.14) numerically favored dual immunotherapy, these differences were not statistically significant.

Exploratory biomarker analyses suggested that, among patients receiving neoadjuvant nivolumab plus ipilimumab, higher baseline four-gene inflammatory signature scores were associated with improved EFS and pCR, whereas this association was not observed with chemotherapy alone [19]. These findings support further investigation of CTLA-4 blockade and other immunotherapeutic combinations in resectable NSCLC. Additional strategies under clinical investigation include antibody–drug conjugates, therapeutic cancer vaccines, novel immune checkpoint combinations, targeted therapies, and integration of radiotherapy or tumor-directed ablative approaches. Whether these strategies can increase pCR rates and, more importantly, translate into improved EFS and OS requires prospective evaluation.

### 5.5. Squamous Cell Histology 

Patients with resectable squamous NSCLC also derive benefit from perioperative ICI-based therapy, although subgroup findings have differed across trials. In CheckMate-816, the EFS benefit of neoadjuvant nivolumab plus chemotherapy appeared attenuated in the squamous histology subgroup compared with nonsquamous disease. In contrast, CheckMate-77T demonstrated an EFS benefit with perioperative nivolumab plus chemotherapy in patients with squamous histology [27]. Similarly, subgroup analyses from KEYNOTE-671 and NEOTORCH demonstrated EFS benefit with perioperative pembrolizumab and toripalimab, respectively, among patients with squamous histology [51,128].

These findings raise the hypothesis that the adjuvant component of perioperative therapy may contribute additional benefit in some patients with squamous NSCLC following neoadjuvant chemoimmunotherapy. However, this cannot be established from cross-trial comparisons, and no randomized trial has directly isolated the incremental effect of adjuvant ICI in this subgroup. Overall, available data support the efficacy of perioperative chemoimmunotherapy in resectable squamous NSCLC and do not support excluding patients from an ICI-based perioperative approach on the basis of squamous histology alone.

## 6. Conclusions and Future Directions

Precision oncology in NSCLC has expanded from advanced disease into early-stage disease treated with curative intent. Successful implementation of precision perioperative therapy requires close multidisciplinary collaboration among medical oncologists, thoracic surgeons, radiation oncologists, pathologists, radiologists, and molecular diagnostic teams. Integration of clinical stage, resectability, molecular profile, and treatment eligibility provides a practical framework for selecting contemporary perioperative strategies for resectable NSCLC (Figure 3). For NSCLC patients with AGAs, adjuvant targeted therapies have substantially reduced the risk of disease recurrence in NSCLC harboring *EGFR* classical mutations, *ALK* fusions, and *RET* fusions. While OS data are not mature for alectinib (ALINA) and selpercatinib (LIBRETTO-432), adjuvant osimertinib (ADAURA) has demonstrated a significant OS benefit. For patients without classic *EGFR* mutations or *ALK* alterations, neoadjuvant or perioperative PD-(L)1 blockade combined with platinum-based chemotherapy has become a standard of care for appropriately selected patients with resectable stage II–III NSCLC. However, the optimal perioperative management of tumors harboring other oncogenic drivers, including ROS1 and NRG1 fusions, remains uncertain. Although targeted therapies are available for selected patients with these alterations in advanced disease, their roles in the perioperative setting have not been established. These developments highlight the importance of comprehensive molecular profiling, ideally incorporating both DNA- and RNA-based next-generation sequencing, to guide treatment selection and identify patients for genotype-directed clinical trials. 

Selection of perioperative treatment should be individualized according to disease stage, nodal involvement, resectability, histology, PD-L1 expression, molecular profile, and patient-specific factors. Differences in eligibility criteria, treatment regimens, and TNM staging systems among pivotal trials must also be considered when translating trial results into clinical practice. Important challenges remain in defining the optimal sequencing of systemic therapy and surgery, identifying patients who benefit from postoperative treatment, and determining the clinical effectiveness and cost-effectiveness of increasingly complex perioperative strategies. 

Future strategies should move beyond a uniform perioperative approach toward biomarker- and response-adapted treatment. Integration of pathologic response, ctDNA-defined MRD assessment, and other emerging biomarkers may enable postoperative therapy to be escalated for patients at high risk of recurrence while allowing treatment to be safely de-escalated in those with favorable responses. Such approaches may be particularly important in determining the incremental value and optimal duration of postoperative therapy after effective neoadjuvant treatment. Continued multidisciplinary collaboration, risk-adapted treatment selection, prospective biomarker validation, and rigorous assessment of cost-effectiveness will be essential to realizing the full potential of precision perioperative therapy and improving long-term outcomes for patients with resectable NSCLC.

## Figures and Tables

**Table 1:** **Selected phase II–III trials of neoadjuvant, perioperative, adjuvant, and consolidation immunotherapy in early-stage and locally advanced NSCLC.** Selected prospective trials evaluating ICIs alone or in combination with chemotherapy across resectable and unresectable non-small cell lung cancer (NSCLC). Reported efficacy outcomes include pathologic complete response (pCR), major pathologic response (MPR), disease-free survival (DFS), event-free survival (EFS), progression-free survival (PFS), and overall survival (OS). Hazard ratios (HRs) are reported for the experimental versus control arms unless otherwise specified. Staging reflects the American Joint Committee on Cancer (AJCC) edition used in each study. Molecular eligibility and subgroup results are summarized where clinically relevant. Abbreviations: AJCC, American Joint Committee on Cancer; DFS, disease-free survival; EFS, event-free survival; HR, hazard ratio; MPR, major pathologic response; NSCLC, non-small cell lung cancer; OS, overall survival; pCR, pathologic complete response; PFS, progression-free survival.

Trial Name (NCT or UMIN)	Setting	Immunotherapy	Phase	Regions of Study Participants	Stage (AJCC Edition)	Treatment and Schedule	Molecular Alteration	Key Results	Clinical Significance	References
IMpower010 (NCT02486718)	Adjuvant	Atezolizumab	III	Asia, Europe, North America, Oceania	Completely resected IB–IIIA after adjuvant chemo (7th)	Surgery → platinum-based chemotherapy ×1–4 cycles → atezolizumab up to ×16 (~1 yr)	*EGFR*/*ALK* allowed	Final DFS: All-randomized stage II–IIIA HR 0.83; Stage II–IIIA PD-L1 ≥ 1% HR 0.70; Stage II–IIIA PD-L1 ≥ 50% HR 0.48 Second Interim OS: All-randomized stage II–IIIA HR 0.94; Stage II–IIIA PD-L1 ≥1% HR 0.77; Stage II–IIIA PD-L1 ≥50% HR 0.47	Established adjuvant atezolizumab after platinum-based chemotherapy as a treatment option for resected, PD-L1-positive stage II–IIIA disease, with durable DFS and OS benefits in the PD-L1 ≥50% subgroup	Felip (2021) [30]; Felip (2023) [50]; Felip (2025) [31]
LCMC3 (NCT02927301)	Neoadjuvant, with optional adjuvant	Atezolizumab	II	North America (United States only)	Resectable IB–selected IIIB (8th)	Atezolizumab ×2 → surgery → optional adjuvant chemotherapy ± radiotherapy → optional atezolizumab up to 1 yr	*EGFR*/*ALK* allowed	MPR rate 20%; pCR 6%; 3 yr DFS 72%; 3 yr OS 80%; 4 yr DFS 70%; 4 yr OS 82%	Demonstrated feasibility and antitumor activity of neoadjuvant atezolizumab monotherapy in resectable NSCLC without a survival benefit, supporting further development of perioperative strategies	Chaft (2022) [32]; final manuscript pending; 2024 update provided at WCLC [34]
IMpower030 (NCT03456063)	Perioperative	Atezolizumab	III	Asia, Europe, North America, Oceania, South America	Resectable II–selected IIIB (8th)	Atezolizumab + platinum-based chemotherapy ×4 → surgery → atezolizumab up to ×16 (~1 yr)	*EGFR*/*ALK* excluded	Primary stage IIB–IIIB *EGFR* /*ALK* WT population: mEFS 62.8 vs. 34.9 mo; HR 0.77 (95% CI, 0.58–1.02; *p* = 0.07); pCR 29.6% vs. 8.5%; MPR 53.6% vs. 24.4%	Demonstrated increased pCR/MPR rates but no statistically significant EFS benefit with perioperative atezolizumab plus neoadjuvant chemotherapy	Manuscript pending; 2026 final analysis provided at WCLC [37]
PACIFIC (NCT02125461)	Consolidation	Durvalumab	Unresectable III (7th)	Asia, Europe, North America, Oceania, South America	Unresectable stage III without progression after chemoradiation (7th)	Platinum-based concurrent chemoradiation → durvalumab up to 1 yr	*EGFR*/*ALK* allowed, benefit uncertain in these subgroups	mPFS 16.9 vs. 5.6 months (HR 0.55); mOS 47.5 vs. 29.1 months (HR 0.72); five-year OS 42.9% vs. 33.4%	Established consolidation durvalumab as the standard of care for unresectable stage III disease without progression after definitive chemoradiation	Antonia (2017) [39]; Spigel (2022) [42]
SAKK 16/14 (NCT02572843)	Perioperative	Durvalumab	II	Europe (Switzerland only)	Resectable stage IIIA, T1–3N2M0 (7th)	Cisplatin/docetaxel ×3 → durvalumab ×2 → surgery → durvalumab up to ×26 (~1 yr)	*EGFR*/*ALK* allowed	1 yr EFS 73%; MPR 62%, pCR 18%; 5 yr EFS 46%, OS 66%; median OS not reached	Provided proof-of-concept for combining neoadjuvant chemotherapy with perioperative durvalumab in resectable stage IIIA(N2) disease	Rothschild (2021) [40]; Schmid (2025) [43]
AEGEAN (NCT03800134)	Perioperative	Durvalumab	III	Asia, Europe, North America, Oceania, South America	Resectable stage IIA–IIIB (8th)	Durvalumab + platinum-based chemotherapy ×4 → surgery → durvalumab up to ×12 (~1 yr)	*EGFR*/*ALK* excluded from efficacy analyses	Initial: EFS HR 0.68; pCR 17.2% vs. 4.3%. Updated: EFS HR 0.69 (95% CI, 0.55–0.88); median EFS NR vs. 30.0 mo; interim OS HR 0.89 (95% CI, 0.70–1.14)	Demonstrated that perioperative durvalumab plus neoadjuvant chemotherapy improves EFS and pCR in resectable disease	Heymach (2023) [41]; Heymach (2026) [45]
CheckMate-816 (NCT02998528)	Neoadjuvant	Nivolumab	III	Asia, Europe, North America, Oceania, South America	Resectable IB–IIIA (7th)	Nivolumab + platinum-doublet chemotherapy ×3 → surgery ± postoperative chemotherapy/radiotherapy per institutional practice	*EGFR*/*ALK* excluded	mEFS 31.6 vs. 20.8 mo (HR 0.63); pCR 24% vs. 2.2%; 5-year OS 65.4% vs. 55.0% (HR 0.72)	First phase III trial to establish neoadjuvant chemoimmunotherapy as a standard treatment for resectable disease, demonstrating significant improvements in EFS, pCR, and OS	Forde (2022) [17]; Forde (2025) [18]
NADIM II (NCT03838159)	Perioperative	Nivolumab	II	Europe (Spain only)	Resectable stage IIIA–IIIB (7th)	Nivolumab + carboplatin/paclitaxel ×3 → surgery → nivolumab ×6 mos after R0 resection	*EGFR*/*ALK* excluded	pCR 37% vs. 7%; 24 mo PFS 67.2% vs. 40.9% (HR 0.47); 24 mo OS 85.0% vs. 63.6% (HR 0.43)	Provided randomized evidence that perioperative nivolumab plus chemotherapy improves pCR and OS in resectable stage III NSCLC	Provencio (2023) [20]
CheckMate-77T (NCT04025879)	Perioperative	Nivolumab	III	Asia, Europe, North America, Oceania, South America	Resectable IIA–IIIB (8th)	Nivolumab + platinum-doublet chemotherapy ×4 → surgery → nivolumab up to ×13 (~1 yr)	*EGFR*/*ALK* excluded	pCR 25.3% vs. 4.7%; mEFS 46.6 vs. 16.9 mo (HR 0.61); 30 mo OS 78% vs. 72% (HR 0.85; interim)	Demonstrated efficacy of perioperative nivolumab plus chemotherapy and supported its approval in resectable NSCLC; the incremental benefit of adjuvant nivolumab after neoadjuvant chemoimmunotherapy remains uncertain	Cascone (2024) [27]; Cascone (2025; ASCO abstract LBA8010; full manuscript pending) [23]
KEYNOTE-091 (PEARLS) (NCT02504372)	Adjuvant	Pembrolizumab	III	Asia, Europe, North America, Oceania, South America	Completely resected IB–IIIA (7th)	Surgery ± adjuvant platinum-based chemotherapy (up to 4 cycles) → pembrolizumab up to ×18 (~1 yr)	*EGFR*/*ALK* allowed	mDFS 53.8 vs. 43.0 mo (HR 0.81); mOS data remain immature (prior interim OS HR 0.87, 95% CI 0.67–1.15)	Demonstrated sustained DFS benefit with adjuvant pembrolizumab in PD-L1-unselected resected NSCLC; a definitive OS benefit has not yet been established	O’Brien (2022) [25]; Besse (2023; ESMO-IO abstract 120MO; final DFS analysis; full manuscript pending) [26]
KEYNOTE-671 (NCT03425643)	Perioperative	Pembrolizumab	III	Asia, Europe, North America, Oceania, South America	Resectable II–IIIB (8th)	Pembrolizumab + cisplatin-based chemotherapy ×4 → surgery → pembrolizumab up to ×13	*EGFR*/*ALK* included but small sample size	pCR 18.1% vs. 4.0%; 5-year EFS 49.9% vs. 26.5% (HR 0.58); 5-year OS 64.6% vs. 53.6% (HR 0.74)	First phase III perioperative immunotherapy trial to demonstrate a significant OS benefit, establishing perioperative pembrolizumab plus chemotherapy as a standard treatment strategy	Wakelee (2023) [28]; Wakelee (2026) [29]
NEOTORCH (NCT04158440)	Perioperative	Toripalimab	III	Asia (China only)	Resectable II–IIIB; predominantly squamous; 2024 interim efficacy analysis limited to stage III	Toripalimab + platinum-based chemotherapy ×3 → surgery → toripalimab + chemotherapy ×1 → toripalimab up to ×13	*EGFR*/*ALK* excluded in nonsquamous tumors	EFS HR 0.40; pCR 24.8% vs. 1.0%; MPR 48.5% vs. 8.4%; interim OS HR 0.62	Demonstrated EFS and pCR benefits with perioperative toripalimab plus chemotherapy in resectable stage III disease; study population was predominantly squamous	Lu (2024) [51]

**Table 2:** Overview of select phase II–III trials of neoadjuvant, adjuvant, and perioperative targeted therapy for resectable NSCLC.

Trial Name (NCT or UMIN)	Setting	Targeted Therapy	Phase	Regions of Study Participants	Stage (AJCC Edition)	Treatment and Schedule	Molecular Alteration	Key Results	Clinical Significance	References
ADAURA (NCT02511106)	Adjuvant	Osimertinib	III	Asia, Europe, North America, South America, Oceania	Completely resected IB–IIIA (7th)	Surgery ± platinum-based chemotherapy → osimertinib 80 mg daily for up to 3 yr vs. placebo	*EGFR* exon 19 deletion or exon 21 L858R	Stage II–IIIA: DFS HR 0.23; 4 yr DFS 70% vs. 29%; CNS DFS HR 0.24; 8 yr OS 74% vs. 58% (HR 0.53). Overall stage IB–IIIA: 8 yr OS 79% vs. 64% (HR 0.52)	Established adjuvant osimertinib as a standard of care and demonstrated both OS and CNS-recurrence benefits	Wu (2020) [66]; Tsuboi (2023) [67]; Wu (2026) [68]
ALINA (NCT03456076)	Adjuvant	Alectinib	III	Africa, Asia, Europe, Middle East, North America, Oceania	Completely resected IB (≥4 cm)–IIIA (7th)	Surgery → alectinib 600 mg twice daily for 2 yr vs. platinum-based chemotherapy ×4 cycles	*ALK* fusion/rearrangement	4 yr DFS 75.5% vs. 47.0% (HR 0.35); 4 yr CNS DFS 90.4% vs. 76.1% (HR 0.37); 4 yr OS 98.4% vs. 92.4% (HR 0.40; OS immature)	Established adjuvant alectinib as the first targeted standard for resected *ALK*-positive NSCLC, with substantial systemic and CNS DFS benefit versus chemotherapy	Wu (2024) [69]; Dziadziuszko (2025) [70]; Barlesi (2026) [71]
ELEVATE (NCT05341583)	Adjuvant	Ensartinib	III	Asia (China only)	Completely resected IB–IIIB (8th)	Surgery → protocol-appropriate adjuvant platinum-based chemotherapy → ensartinib 225 mg daily for 24 mo vs. placebo	*ALK* fusion/rearrangement	Stage II–IIIB: 2 yr DFS 86.4% vs. 53.5% (HR 0.20); overall stage IB–IIIB: 2 yr DFS 87.3% vs. 57.2% (HR 0.20); CNS DFS HR 0.22; brain recurrence 3.6% vs. 12.4%; OS immature	Demonstrated substantial incremental benefit from adjuvant *ALK* inhibition after chemotherapy; OS data remain immature	Yue (2026) [72]
LIBRETTO-432 (NCT04819100)	Adjuvant	Selpercatinib	III	Asia, Europe, North America, Oceania, South America	Stage IB–IIIA after definitive curative-intent therapy (8th)	Definitive surgery or radiotherapy ± adjuvant systemic therapy → selpercatinib 160 mg twice daily for up to 3 yr vs. placebo	*RET* fusion	Stage II–IIIA: 2 yr EFS 92% vs. 61% (HR 0.17); median EFS NR vs. 31.8 mo Stage IB–IIIA: 2 yr EFS 94% vs. 70% (HR 0.17); OS immature	First randomized phase III evidence supporting RET inhibition after definitive curative-intent therapy for early-stage RET fusion-positive NSCLC	Wu (2026) [73]
ADJUVANT/CTONG1104 (NCT01405079)	Adjuvant	Gefitinib	III	Asia (China only)	Completely resected II–IIIA (N1–N2) (7th)	Gefitinib 250 mg daily for 24 mo vs. cisplatin/vinorelbine ×4 cycles	*EGFR* exon 19 deletion or exon 21 L858R	Median DFS 30.8 vs. 19.8 mo; DFS HR 0.56; 5 yr DFS 22.6% vs. 23.2%; median OS 75.5 vs. 62.8 (HR 0.92); 5 yr OS 53.2% vs. 51.2%;	First-generation *EGFR* TKI with an early DFS advantage without durable DFS or OS improvement	Zhong (2018) [74]; Zhong (2021) [75]
EVAN (NCT01683175)	Adjuvant	Erlotinib	II	Asia (China only)	Completely resected stage IIIA (7th)	Erlotinib 150 mg daily for 2 yr vs. cisplatin/vinorelbine ×4 cycles	*EGFR* exon 19 deletion or exon 21 L858R	2 yr DFS 81.4% vs. 44.6%; median OS 84.2 vs. 61.1 mo; 5 yr OS 84.8% vs. 51.1%	Provided an early randomized signal that adjuvant *EGFR* inhibition could improve outcomes in high-risk resected stage IIIA disease	Yue (2018) [76]; Yue (2022) [77]
EVIDENCE (NCT02448797)	Adjuvant	Icotinib	III	Asia (China only)	Completely resected II–IIIA (7th)	Icotinib 125 mg three times daily for 2 yr vs. cisplatin-based chemotherapy ×4 cycles	*EGFR* exon 19 deletion or exon 21 L858R	Median DFS 47.0 vs. 22.1 mo (HR 0.36); 3 yr DFS 63.9% vs. 32.5%; OS immature (HR 0.91)	Confirmed a marked DFS benefit with a first-generation *EGFR* TKI, although it did not establish a global standard comparable with osimertinib	He (2021) [78]
IMPACT WJOG6410L (UMIN000006252)	Adjuvant	Gefitinib	III	Asia (Japan only)	Completely resected II–IIIA (7th)	Gefitinib 250 mg daily for 2 yr vs. cisplatin/vinorelbine ×4 cycles	*EGFR* exon 19 deletion or exon 21 L858R	Median DFS 35.9 vs. 25.1 mo; DFS HR 0.92; 5 yr OS 78.0% vs. 74.6% (HR 1.03)	Showed early separation followed by crossing of the DFS curves, with no significant durable DFS or OS benefit	Tada (2021) [79]
NeoADAURA (NCT04351555)	Neoadjuvant	Osimertinib ± chemotherapy	III	Asia, Europe, North America, Oceania, South America	Resectable II–IIIB (8th)	Osimertinib + platinum/pemetrexed ×3 cycles or osimertinib alone for ≥9 wk vs. placebo + chemotherapy → surgery; adjuvant osimertinib permitted	*EGFR* exon 19 deletion or exon 21 L858R	MPR 26%, 25%, and 2%; pCR 4%, 9%, and 0%, respectively; EFS immature	First reported randomized phase III trial of neoadjuvant targeted therapy; osimertinib with or without chemotherapy significantly increased MPR versus chemotherapy alone without compromising surgical feasibility, although EFS remains immature	He (2025) [80]
Phase II neoadjuvant osimertinib study (NCT03433469)	Neoadjuvant	Osimertinib	II	North America (United States only)	Resectable I–IIIA (7th)	Osimertinib 80 mg daily for up to two 28-day cycles → surgery	*EGFR* exon 19 deletion or exon 21 L858R	MPR 14.8%; pCR 0%; ORR 52%; median DFS 40.9 mo; 24 of 27 patients underwent surgery	Demonstrated the feasibility and surgical safety of short-course neoadjuvant osimertinib, although the prespecified MPR endpoint was not met and 11% did not proceed to surgery	Blakely (2024) [81]
EMERGING-CTONG1103 (NCT01407822)	Perioperative	Erlotinib	II	Asia (China only)	Resectable stage IIIA-N2 (7th)	Erlotinib for 42 days → surgery → erlotinib for up to 12 mo vs. gemcitabine/cisplatin ×2 before and ×2 after surgery	*EGFR* exon 19 deletion or exon 21 L858R	ORR 54.1% vs. 34.3% (primary endpoint not met); MPR 9.7% vs. 0%; pCR 0% in both arms; median PFS 21.5 vs. 11.4 mo (HR 0.36); median OS 42.2 vs. 36.9 mo (HR 0.83); 5 yr OS 40.8% vs. 27.6%	Provided proof-of-concept for perioperative *EGFR* inhibition but was limited by small size and failure to meet its primary endpoint	Zhong (2019) [82]; Zhong (2023) [83]
NORA (NCT04816838)	Perioperative	Osimertinib	II	Asia (South Korea only)	Resectable IA–IIIA (8th)	Osimertinib 80 mg daily ×2 cycles → surgery → osimertinib for up to 3 yr	*EGFR* exon 19 deletion or exon 21 L858R	ORR 44% (primary endpoint not met); MPR 24%; pCR 0%; R0 resection 100%; median DFS NR at 31 mo	Supported the feasibility of a short neoadjuvant course followed by prolonged adjuvant osimertinib, but was limited by its small single-center design	Lee (2025) [84]
ALNEO (NCT05015010)	Perioperative	Alectinib	II	Europe (Italy only)	Potentially resectable stage IIIA (8th)	Alectinib for approximately 8 wk → surgery → alectinib for up to 24 mo	*ALK* fusion/rearrangement	MPR 46%; pCR 12%; ORR 67%; 85% underwent surgery; R0 resection 86%; median EFS and OS not reached	Provided a strong phase II efficacy and feasibility signal for perioperative *ALK* inhibition; randomized confirmation is needed	Leonetti (2025) [85], final manuscript pending
NAUTIKA1 (NCT04302025)	Perioperative	Molecularly matched targeted therapy	II	North America (United States only)	Resectable IB–IIIA and selected IIIB (8th)	Protocol-specific matched neoadjuvant therapy for approximately 8 wk → surgery → adjuvant chemotherapy as indicated and matched therapy, generally for up to approximately 2 yr	*ALK*, *ROS1*, *NTRK1/2/3*, *BRAF V600*, *RET*, and *KRAS* G12C	*ALK* cohort (N = 48): MPR 55.8%; pCR 18.6%; ORR 60.0%; R0 resection 95%; 2 yr EFS 94%, DFS 96%, OS 97% KG12C cohort (N = 20): MPR 42% (8/19); pCR 16% (3/19); ORR 55% (11/20); R0 resection 100% (19/19); no PD; no AE-related surgical delays	Demonstrated clinically meaningful pathologic responses and surgical feasibility with perioperative alectinib in resectable *ALK*-positive NSCLC; results from other molecular cohorts remain ongoing	Lee (2023) [86]; Lee (2026) [87]

**Table 3:** Surgical outcomes of resected NSCLC following neoadjuvant chemoimmunotherapy. This table summarizes surgical outcomes in patients with resectable NSCLC treated with neoadjuvant chemotherapy plus immune checkpoint inhibitors across selected clinical studies. Key outcomes include surgical resection rates, completeness of resection (R0), surgical approaches, extent of pulmonary resection, conversion to open surgery, postoperative complications, and perioperative mortality, where reported. Differences in study populations, treatment regimens, surgical practices, and outcome definitions should be considered when interpreting comparisons across studies. Abbreviations: MIS, minimally invasive surgery; NR, not reported; NSCLC, non-small cell lung cancer; R0, microscopically margin-negative resection; VATS, video-assisted thoracoscopic surgery; RATS, robotic-assisted thoracoscopic surgery.

Trial	Surgery Performed	R0 Resection	N2/Resectability	Operative Approach/Extent	Conversion/Technical Difficulty	Postoperative Morbidity/Mortality	Delay/Cancellation
CheckMate-816	83.2% vs. 75.4%	83% vs. 78% of operated	Stage IB–IIIA; N2-specific operative details not in cited surgical abstract	Lobectomy 77% vs. 61%; pneumonectomy 17% vs. 25%; MIS 30% vs. 22%	MIS-to-open conversion 11% vs. 16%; median operative time 184 vs. 217 min	AEs: 2 vs. 2; AE-related delay: 6 vs. 9	Surgery canceled for progression: 12 vs. 17 patients
CheckMate-77T	78% vs. 77%	89% vs. 90% of operated	N2 subgroup: surgery 77% vs. 73%; R0 86% vs. 86%	Pneumonectomy 9% vs. 14% overall; N2 subgroup 1% vs. 14%	NR	Grade 3–4 surgery-related AEs 12% vs. 12%; grade 5 2% vs. 1%	NR
KEYNOTE-671	82.1% vs. 79.4%	92.0% vs. 84.2% of operated	Stage II–IIIB; nodal downstaging 38.9% vs. 28.4%	Lobectomy 78.8% vs. 75.1%; pneumonectomy 11.4% vs. 12.3%	NR	Surgery-related deaths ≤ 30 days: 6 vs. 2; other complication rates NR	Surgical delay 4.9% vs. 7.6%
AEGEAN	80.6% vs. 80.7% underwent; 77.6% vs. 76.7% completed	94.7% vs. 91.3% of operated	N2→N0 downstaging 47.3% vs. 40.2%	MIS 49.2% vs. 47.0%; open 49.2% vs. 50.7%; lobectomy 88.1% vs. 85.4%	Conversion NR in abstract; median last dose to surgery 34 days both arms	Surgical complications 59.1% vs. 60.1% (mostly grade 1–2)	Delayed surgery 17.3% vs. 22.2% of those undergoing surgery
IMpower030 (WCLC 2026 final analysis)	NR (surgery cancellation reported separately; not equivalent to surgery completion)	NR (R0 percentage not verified)	Resectable stage II–IIIA and selected IIIB (T3N2); *EGFR*/*ALK* wild-type efficacy population	NR (lobectomy, pneumonectomy, minimally invasive/open proportions not verified)	NR	No new safety signal reported; specific postoperative morbidity/mortality NR	Surgery cancelled: 11.1% vs. 10.3% (atezolizumab + chemotherapy vs. placebo + chemotherapy); reasons and surgery delays NR

## Data Availability

No new data were created or analyzed in this study. Data sharing is not applicable to this article.

## List of Abbreviations

AGA	actionable genomic alteration
CI	confidence interval
CNS	central nervous system
CT	chemotherapy
DFS	disease-free survival
EFS	event-free survival
HR	hazard ratio
ICI	immune checkpoint inhibitor
IHC	immunohistochemistry
mEFS	median event-free survival
MRD	minimal residual disease
NSCLC	non-small cell lung cancer
OR	odds ratio
OS	overall survival
PCR	polymerase chain reaction
PFS	progression-free survival
R0	complete resection
RFS	recurrence-free survival
RR	relative risk
TCR	T-cell receptor
TKI	tyrosine kinase inhibitor
TLS	tertiary lymphoid structures
TPS	tumor proportion score
TME	tumor microenvironment
Treg	regulatory T cell
